# Identification of susceptibility loci using a novel murine model for triple-negative breast cancer

**DOI:** 10.1093/g3journal/jkaf238

**Published:** 2025-10-10

**Authors:** Minjeong Kim, Logan G McGrath, Zeid T Mustafa, Samson Eugin Simon, Naveed Pervaiz, Emily W Grey, Sydney C Joseph, Emily Korba, Sandesh J Marathe, Margaret S Bohm, Arvind V Ramesh, Sidharth S Mahajan, Casey J Bohl, Pjotr Prins, Robert W Read, Jeremiah R Holt, D Neil Hayes, Lu Lu, Robert W Williams, Laura M Sipe, David G Ashbrook, Liza Makowski

**Affiliations:** Division of Hematology and Oncology, Department of Medicine, College of Medicine, The University of Tennessee Health Science Center (UTHSC), Memphis, TN 38163, United States; Division of Hematology and Oncology, Department of Medicine, College of Medicine, The University of Tennessee Health Science Center (UTHSC), Memphis, TN 38163, United States; Division of Hematology and Oncology, Department of Medicine, College of Medicine, The University of Tennessee Health Science Center (UTHSC), Memphis, TN 38163, United States; Division of Hematology and Oncology, Department of Medicine, College of Medicine, The University of Tennessee Health Science Center (UTHSC), Memphis, TN 38163, United States; Division of Hematology and Oncology, Department of Medicine, College of Medicine, The University of Tennessee Health Science Center (UTHSC), Memphis, TN 38163, United States; Division of Hematology and Oncology, Department of Medicine, College of Medicine, The University of Tennessee Health Science Center (UTHSC), Memphis, TN 38163, United States; Division of Hematology and Oncology, Department of Medicine, College of Medicine, The University of Tennessee Health Science Center (UTHSC), Memphis, TN 38163, United States; Division of Hematology and Oncology, Department of Medicine, College of Medicine, The University of Tennessee Health Science Center (UTHSC), Memphis, TN 38163, United States; Division of Hematology and Oncology, Department of Medicine, College of Medicine, The University of Tennessee Health Science Center (UTHSC), Memphis, TN 38163, United States; UTHSC Center for Cancer Research, College of Medicine, UTHSC, Memphis, TN 38163, United States; Department of Microbiology, Immunology, and Biochemistry, UTHSC, Memphis, TN 38163, United States; Division of Hematology and Oncology, Department of Medicine, College of Medicine, The University of Tennessee Health Science Center (UTHSC), Memphis, TN 38163, United States; Division of Hematology and Oncology, Department of Medicine, College of Medicine, The University of Tennessee Health Science Center (UTHSC), Memphis, TN 38163, United States; Department of Genetics, Genomics, and Informatics, UTHSC, Memphis, TN 38163, United States; Department of Genetics, Genomics, and Informatics, UTHSC, Memphis, TN 38163, United States; Department of Genetics, Genomics, and Informatics, UTHSC, Memphis, TN 38163, United States; Department of Genetics, Genomics, and Informatics, UTHSC, Memphis, TN 38163, United States; Division of Hematology and Oncology, Department of Medicine, College of Medicine, The University of Tennessee Health Science Center (UTHSC), Memphis, TN 38163, United States; UTHSC Center for Cancer Research, College of Medicine, UTHSC, Memphis, TN 38163, United States; Department of Genetics, Genomics, and Informatics, UTHSC, Memphis, TN 38163, United States; UTHSC Center for Cancer Research, College of Medicine, UTHSC, Memphis, TN 38163, United States; Department of Genetics, Genomics, and Informatics, UTHSC, Memphis, TN 38163, United States; UTHSC Center for Cancer Research, College of Medicine, UTHSC, Memphis, TN 38163, United States; Department of Genetics, Genomics, and Informatics, UTHSC, Memphis, TN 38163, United States; Department of Biology, University of Mary Washington, Fredericksburg, VA 22401, United States; UTHSC Center for Cancer Research, College of Medicine, UTHSC, Memphis, TN 38163, United States; Department of Genetics, Genomics, and Informatics, UTHSC, Memphis, TN 38163, United States; Division of Hematology and Oncology, Department of Medicine, College of Medicine, The University of Tennessee Health Science Center (UTHSC), Memphis, TN 38163, United States; UTHSC Center for Cancer Research, College of Medicine, UTHSC, Memphis, TN 38163, United States; Department of Microbiology, Immunology, and Biochemistry, UTHSC, Memphis, TN 38163, United States; Department of Genetics, Genomics, and Informatics, UTHSC, Memphis, TN 38163, United States

**Keywords:** systems genetics, QTL, eQTL, hybrid, gene variant, tumor microenvironment, antitumor immunity

## Abstract

Triple-negative breast cancer (TNBC) is the deadliest subtype of breast cancer (BC) with few targeted therapies. To identify novel genetic modifiers of TNBC, we created a murine model incorporating high levels of genetic and phenotypic diversity. C3(1)-T-antigen (“C3Tag”) mice, which develop spontaneous basal-like TNBC tumors, were systematically crossed with a large set of sequenced BXD recombinant inbred strains to produce isogenic hybrids segregating for C3Tag. The severity of TNBC traits including tumor latency, multiplicity, and survival was highly variable and heritable. We mapped modifiers of TNBC and identified loci on chromosomes 16 and 10 associated with tumor multiplicity and latency, respectively. Candidate genes were prioritized including a lysosomal enzyme involved in cell proliferation, *Gns*; tumor suppressor *Rassf3*; and Rab-modifying *Tbc1d30*. In tumors from BC patients, higher GNS, RASSF3, and TBC1D30 expression associated with poor overall survival. In sum, we developed a clinically relevant, BXD-BC model which provides robust genetic heterogeneity enabling the identification of conserved modifiers and mediators of BC.

## Teaser

A novel mouse model was developed for a target audience of cancer genetics researchers. Cancer-prone C3(1)-T-antigen mice were crossed with genetically diverse BXD recombinant inbred strains. This clinically relevant model is useful because it has publicly available data through GeneNetwork. Findings have uncovered genes to guide future TNBC treatments. Hybrids showed significantly heritable variation in tumor latency, multiplicity, and survival. Genetic modifiers impacting tumor traits were mapped with loci identified on chromosomes 16 and 10 linked to BC tumor onset and growth. Key conserved genes-GNS, RASSF3, and TBC1D30—correlated with poor survival in BC patients.

## Introduction

Breast cancer (BC) is a significant global health concern, encompassing a heterogeneous group of malignancies that vary in their molecular characteristics, clinical behavior, and treatment responses. BC is the second most common cause of death in women worldwide (Sung et al. 2021). While deleterious germline mutations in genes including *MDM2*, *NUP107*, *HER2*, *BRCA1*, and *BRCA2* are commonly used in genetic diagnostic tests, known mutations only account for ∼10% of BC cases, emphasizing the complexity and limitations of diagnosis and risk prediction (Cancer Genome Atlas Network 2012; Coignard et al. 2021; Hopper et al. 2024; Li et al. 2024). Triple-negative BC (TNBC) represents around 15% to 20% of all BCs and is associated with aggressive clinical outcomes, such as early recurrence and unique metastatic patterns. TNBC risk factors include obesity, alcohol exposure, and underlying genetics (e.g. *BRCA1/2* genes) (da Costa Nunes et al. 2024; Head et al. 2024; Li et al. 2024). There are not yet effective individualized approaches to TNBC prevention or therapeutics. Due to these limitations, patients with TNBC experience elevated recurrence and metastasis and poorer overall survival (OS) relative to patients with other BC subtypes (Kohler et al. 2015; Zagami and Carey 2022; Capuozzo et al. 2024). This challenge can be met by increasing the translatability and power of preclinical models to both define germline modifiers of TNBC and ultimately test the efficacy of interventions.

To identify genetic modifiers of TNBC phenotypes that could improve future risk assessment or advance therapeutics for BC patients, we developed a preclinical model with highly variable and heritable variation in TNBC by crossing 2 well-established murine models—the C3(1)-T-antigen (“C3Tag”) mouse—with the largest fully sequenced family of mice, the BXD (Sasani et al. 2022). The C3Tag strain is a model that resembles the human basal-like TNBC subtype. C3Tag mice recapitulate the common loss-of-function mutations in the tumor suppressor genes retinoblastoma (*RB1*) and tumor protein 53 (*TP53)* to induce spontaneous BC tumors (Maroulakou et al. 1994; Herschkowitz et al. 2007, 2008; Cozzo et al. 2016; Usary et al. 2016). We and others have reported that the C3Tag is a highly penetrant model: hemizygotes progressively develop intraepithelial neoplasia after ∼8 weeks, ductal hyperplasia similar to ductal carcinoma in situ (DCIS) by ∼3 months of age, and finally adenocarcinoma by 5 to 6 months of age in 100% of the females (Herschkowitz et al. 2007; Sundaram et al. 2013; Sundaram, Freemerman, et al. 2014; Sundaram, Le, et al. 2014; Cozzo et al. 2016; Qin et al. 2016). To create a novel preclinical BC model, C3Tag mice were intercrossed into the BXD family. The BXD family is the oldest set of recombinant inbred strains of mice (∼120 fully inbred sub-strains) generated starting in the 1970s by crossing C57BL/6J (“B”) and DBA/2J (“D”) strains—ie “BXD” (Williams et al. 2001; Peirce et al. 2004; Ashbrook et al. 2021). In our work, we take advantage of the dominance of the C3Tag variants and crossed male carriers to 28 BXD strains.

Female BXD-BC F1 progeny developed tumors across all mammary glands with high penetrance and variance across F1 hybrids. We evaluated tumor latency, multiplicity, and survival. To identify genetic drivers of these traits in mice, we mapped multiple quantitative trait loci (QTLs) and identified high-priority protein-coding candidates. We exploited publicly available expression-QTL (eQTL) data sets for the BXD family to link phenotypes with genes of interest.

To translate these mouse findings into the human context, we next performed comparative analysis using human phenome-wide association studies (PheWAS) of homologous chromosomal regions. This approach enabled us to prioritize strong candidates, particularly RASSF3, a tumor suppressor that has multiple intron variants linked to BC and that also modulated the expression of 2 other candidate genes—TBC1D30 and GNS. Finally, in BC patients, OS was improved with greater tumor RASSF3, TBC1D30, and GNS combined expression. In sum, using cutting-edge systems genetics in the BXD-BC model, we identified conserved genetic modifiers of BC phenotypic variation that will be examined as potential molecular targets for novel therapeutic approaches or utilized as biomarkers of risk or response to therapy.

## Materials and methods

### Reagents

All reagents were obtained from Sigma-Aldrich (St. Louis, Missouri, United States) unless otherwise noted.

### Animals

**Fig. 1.**
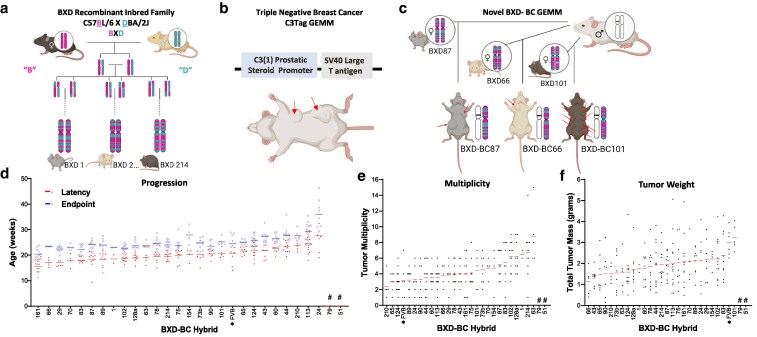
Novel BXD-BC hybrids were generated by crossing C3Tag mice to various hybrids from the BXD recombinant inbred family that demonstrate variability in latency, endpoint, tumor multiplicity, and tumor mass. a) The schema provided demonstrates how C57BL/6J “B” and DBA/2J “D” parents were crossed (“X”) to create BXD mice. The F2 progeny of each BXD cross were iteratively inbred by sibling mating 20 to 100 times, with an isogenic unique mosaic of *B* and *D* alleles labeled as BXD#: BXD1, BXD2…BXD214. The coat colors of the BXD vary as crosses of the B (black) and D (brown) strains. b) C3(1)-T-antigen (“C3Tag”) mice were purchased from The Jackson Laboratory and are well-established models of human basal-like TNBC. The C3Tag is in the FVB/N background, which is a white coat. c) Male mice transgenic for T-antigen were crossed into females of various strains of the BXD recombinant inbred family. C3Tag-BXD F1s breast cancer (BC) models were termed “BXD-BC” followed by the BXD dam's strain number. Tumors develop spontaneously with latency, number, and size of tumors varying both within and between genetic backgrounds (red arrows). d to f) Tumor phenotypes were ranked by mean with individuals represented as data points per mouse. The BXD strain used in each cross is on the *x* axis. The parent C3Tag FVB/N strain is indicated as “FVB” with an “*” asterisk. d) Tumor latency (red circle) and endpoint (blue “x”) are shown with tumor progression defined from age in weeks at latency to endpoint. e) Tumor multiplicity (number of tumors/mouse). f) Tumor weight of all tumors was summed (g) per mouse. “#” denotes F1s with no tumor development after 12 months.

Studies were performed in accordance with the guidelines of the Institutional Animal Care and Use Committee (IACUC) at the University of Tennessee Health Science Center (UTHSC, Animal Welfare Assurance Number A3325-01) and following the National Institutes of Health Guide for the Care and Use of Laboratory Animals. All animals were housed in a temperature-controlled facility with a 12 h light/dark cycle and ad libitum access to food and water. Mice were housed at UTHSC in the same animal facility to minimize external impacts. BXD nulliparous females were supplied from the mouse colony of the Center for Integrative and Translational Genomics (CITG). The BXD recombinant inbred family has been studied for decades with each strain inbred for up to 100 generations to become isogenic, reviewed in detail by Ashbrook et al. (*2*021) The BXD family has been used to identify genes in longevity, vision, development, and behavior (Geisert and Williams 2020; Roy et al. 2021; Morel et al. 2024; Rajan et al. 2025), with this manuscript the first to examine a genetically driven spontaneous tumor intercross. To allow for unbiased genetic variance of breast cancer onset and progression, strains were randomly selected for inclusion in the study generation of F1 hybrids. C3Tag males were obtained from The Jackson Laboratory (*FVB-Tg(C3-1-TAg) cJeg/JegJ*; RRID: IMSR_JAX:013591). The transgene is maintained on an isogenic FVB/NJ background. The *FVB-Tg(C3-1-TAg*) founder “c” available from The Jackson Laboratory has 6 copies of the T-antigen transgene, which is an oncogene. Mice were bred as transgenic males and wild-type FVB/NJ females purchased from JAX Labs. All mice were maintained on Teklad diet chow (Envigo 7912). Breeders were not included in the study beyond 12 months of age. Sample size power calculations were carried out using the publicly available BXD power application (Sasani et al. 2022), based on qtlDesign (Sen et al. 2007). C3Tag-BXD F1 hybrids were termed “BXD-BC” followed by the BXD strain ID number. A total of 28 BXD strains were used in this study. The creation of the BXD-BC model is represented in Figs. 1a to c. Figures were created in Biorender under the licenses available https://BioRender.com/m7×5b1w and https://BioRender.com/7qkmpt5.

### Tumor latency, progression, and endpoint

The C3Tag mouse was created by Jeff Green at the National Cancer Institute (NCI) using recombinant expression of the simian virus 40 early region transforming sequences under the regulatory control of the rat prostatic steroid binding protein C3(1) gene on the FVB/N background strain (Maroulakou et al. 1994). Latency for female C3Tag in the FVB/N genetic background has a mean of 19 to 20 weeks of age, with tumors arising as early as 11 weeks of age in rare cases depending on the variables tested (Sundaram et al. 2013; Sundaram, Freemerman, et al. 2014; Sundaram, Le, et al. 2014; Cozzo et al. 2016; Qin et al. 2016); thus, palpation for tumors was initiated at 9 weeks of age. BXD-BC mice were palpated across all mammary glands twice weekly since tumors may develop in any mammary gland. Once palpable, tumors were measured by a digital caliper 3 times weekly. The first tumor was called “T1” with subsequent tumors noted as “Tn + 1.” Tumors were allowed to grow until a humane tumor endpoint was reached per IACUC protocol (1 tumor > 2 cm or 3 tumors > 1 cm in diameter). Average tumor burden was calculated as total tumor weight normalized to the total number of tumors (multiplicity) per mouse. Observable TNBC traits were recorded for over 13 phenotypes: age at latency; total tumor volume (mm^3^) at endpoint per mouse; volume, mass, and location of first tumor; total mass of all tumors dissectible (grams) at endpoint per mouse; age at endpoint; survival from latency to endpoint; and location and number of tumors at sacrifice (multiplicity) per mouse.

### Endpoint

Mammary tumors were dissected from every mammary gland and weighed. In mice where no palpable tumors were present, the unaffected inguinal fourth mammary gland was isolated. The T1 tumors or mammary glands were divided and flash frozen or formalin (10%) fixed, and paraffin-embedded (FFPE) for histology. All additional tumors were flash frozen and stored at −80 °C until analyzed.

### Histology and quantification

T1 tumors or unaffected fourth mammary fat pads were cut at 5 µm thickness. FFPE sections were stained with hematoxylin and eosin (H + E) and scanned by a Panoramic 250 Flash III (Thermo Fisher Scientific, Tewksbury, Massachusetts, United States) scanner in the UTHSC Center for Cancer Research Imaging Shared Resource. Veterinary Pathologist Dr. Robert Read, DVM, blindly scored H + E sections of the tumor and surrounding mammary fat pad adipose tissue for hyperplasia, DCIS, and invasion in accordance with our previous work (Qin et al. 2016). Analysis for each trait was averaged from more than 3 randomly selected regions of interest (ROI) in tumor H and E sections, and a score was generated. Mitosis per high-powered field was quantified as a marker of aggressive tumors. Epithelial to mesenchymal transition (EMT)-type histology was scored from 0 to 4 with areas of no pleomorphism to faint to distinct sarcomatous transition. Vascularity and stromal content were scored from 0 to 5 with areas of no presence to mild to scirrhous presence quantified. The necrosis score was calculated by examining each section and noting none to cavitary tumor loss across a 5-point scale.

### Heritability (*h*^2^)

Heritability was calculated for measured traits to determine the proportion of phenotypic variance explained by genetic effects, rather than environmental, technical, or stochastic effects. Narrow-sense heritability (*h^2^*), representing the additive genetic variance component, was estimated as the fraction of variance explained by strain in a one-way ANOVA model (Visscher et al. 2008).

### GeneNetwork

Mean values from each hybrid for all collected phenotypes were uploaded to GeneNetwork (GN) for both mapping and to identify candidates using the deep phenome of the BXD family (Zhou and Stephens 2012; Ashbrook et al. 2015, 2019a, 2019b; Ziebarth and Cui 2017; Prins et al. 2019). GN IDs for the project are noted as BXD_2xxxx, with trait dataset IDs noted in Supplementary Table 1.

### Quantitative trait loci mapping and definition of significance

The mean value per hybrid was calculated to reduce environmental variations, increasing the ability to detect QTLs. The BXD family has been produced in several “epochs,” using both standard F2 recombinant inbred methods and advanced intercross recombinant inbred methods (Ashbrook et al. 2021), leading to both expected and unexpected kinship between BXD types that can introduce bias. Thus, Genome-wide Efficient Mixed Model Association (GEMMA) was used, a linear mixed model-based method to correct for kinship (Zhou and Stephens 2012), which is accessible on GeneNetwork.org. GEMMA methods use a set of 21,056 markers derived from whole genome sequencing (WGS) of the BXD family with data publicly accessible (Sloan et al. 2016; Ashbrook et al. 2022). GEMMA was used to calculate the strength and location of the QTL, determining the −logP value for each marker and identifying the peak position of the QTL, which corresponds to the marker with the highest −logP value. A −logP of > 4 was defined as genome-wide significant, and >3 as suggestive. To identify genes within the QTL, a 1.5 −logP drop interval was applied around the peak position, providing approximately a 95% confidence interval (Manichaikul et al. 2006). All genes between the first marker before and after this interval were noted as potential candidates. To maximize the potential to detect a biologically significant gene in the QTL identified, the candidate genes were prioritized to include only protein-coding. Previous work has demonstrated that this admittedly biased approach is highly effective (Manichaikul et al. 2006; Sasani et al. 2022).

### *Cis*-expression quantitative trait loci (*cis*-eQTL) and candidate gene identification

To identify *cis*-eQTLs, the expression of candidate genes was determined using publicly available BXD transcriptome data in GN in tumor-relevant tissues or cell types (spleen, T-regulatory and T-helper cells) (Alberts et al. 2011; Gibson et al. 2011). A *cis-*eQTL was calculated using GEMMA, defined as an eQTL within a peak ±5 Mb of the cognate gene. Further, to evaluate the significance of the *cis*-eQTL gene association with tumor traits, the Spearman or Pearson correlation was calculated between the tumor traits and all mRNA assays within the spleen (GeneNetwork (GN)_Accession ID 287), T-regulatory cells (GN_Accession ID 122), and T-helper cells (GN_Accession ID 319). GenomeMUSter was used to identify segregating variants within QTL regions (Ball et al. 2024). Genes within the confidence interval were examined for segregating variants predicted to alter splicing or protein function.

### Human phenome-wide association analysis study (PheWAS) translation and survival analysis

Human phenome-wide associations (PheWAS) were conducted using genome-wide association (GWA) summary statistics available online from existing studies to detect gene variants associated with relevant human phenotype associations (e.g. cancer or cancer-associated phenotypes). The human genomic regions syntenic to the mouse QTL were assessed using multiple PheWAS databases including Global Biobank Engine, Gene Atlas, PheWAS Catalog, UK Biobank TOPMed-imputed, Genome-Wide Association Studies (GWAS) Atlas, and BioBank Japan PheWeb (Supplementary Table 2; Denny et al. 2013; Canela-Xandri et al. 2018; Watanabe et al. 2019; Gagliano Taliun et al. 2020; Sakaue et al. 2021). To define the syntenic regions, homologous genes at the start and end of each QTL were defined. The human syntenic region was defined as the start of the first human homologous gene to the end of the last human homologous gene. The web-based survival analysis tool Kaplan–Meier plotter (kmplot.com) database was used to determine the association between OS in basal-like BC patients and RNA-seq candidate gene expression levels (Lanczky and Gyorffy 2021).

### Statistics

For mouse and tumor traits, statistical differences between experimental groups were determined using Kaplan–Meier tumor-free survival analysis, One-way or two-way ANOVA, or Student's *t*-test with Fisher's LSD test for individual comparisons. For body weight, body composition, and tumor volume over time within animals, data were treated as repeated measures. All statistics were performed using statistical software within GraphPad Prism version 10.4.1 (GraphPad Software, Inc., La Jolla, California, United States) or software packages noted and cited above. All data are shown as mean ± standard error of the mean (SEM) unless otherwise noted. KMplot and TNMplot analyses were performed using web-based interfaces (Lanczky and Gyorffy 2021). BXD genetic background ancestry was evaluated using haplotypes from C57BL/6 (B) or DBA2J (D) listed in Supplementary Table 3 across the 28 BXD-BC strains included in this study. For each marker, alleles were coded as B, D, or H (heterozygous). We computed per-chromosome and genome-wide proportions of B and D in R (R Core Team 2023). H markers were handled in 2 ways: split equally between B and D (0.5/0.5) or excluded.

## Results

### Generation of the BXD-BC genetically engineered mouse model

Our study design is presented as a graphical abstract (Supplementary Fig. 1). Specifically, female BXDs (Fig. 1a) were crossed to C3Tag (Fig. 1b) males to generate BXD-BC females (Fig. 1c). We produced a total of 28 sets of BXD-BC F1 progeny using 28 BXD strains (BXD#/Rww × FVB C3Tag F1), as well as a set of FVB/N “FVB” parent C3Tag cases as a useful comparative baseline of disease severity. We produced an average of 8 hybrids of each F1 type, but with a wide range due to differences in breeding performance (Supplementary Table 4). While most strains met the minimum of 4 to 6 F1 hybrids per strain, the BXD-BC1 strain only generated 2 pups by the study endpoint. However, to maximize coverage and mapping resolution, these 2 hybrids were included in the analyses. While low within-strain replication increases the standard error for that strain mean, it does not bias the effect size estimates or mapping accuracy, provided the genotype is retained. All traits are listed as GeneNetwork (GN) ID in Supplementary Table 1 and are publicly available at GeneNetwork.org (Sasani et al. 2022). In the 28 BXD strains, B (51%) and D (49%) alleles were almost equally covered regardless of heterozygous region (Supplementary Table 3).

### BXD-BC tumor phenotypes have significant phenotypic heterogeneity relative to FVB C3Tag mouse

To examine survival as measured by tumor progression, age at latency was recorded along with age at humane (IACUC) endpoint (Fig. 1d). Latency and endpoint varied 1.60- to 1.75-fold from the earliest onset (ie early latency) to the latest onset hybrids (ie delayed latency). The F1s with the *earliest* latency included BXD-BC161, 66, 29, and 70 with aggressive tumor onset before 18 weeks of age. F1s that displayed *delayed* latency included BXD-BC44, 210, 113, and 24 hybrids with tumor onset occurring past 23 weeks of age. Absolute progression to endpoint averaged ∼5 weeks but was highly variable, with some hybrids rapidly progressing to endpoint such as BXD-BC90 at 3.06 weeks until endpoint, compared to others which had a much slower progression including BXD-BC154 and BXD-BC24 with progression times as long as 7.79 and 8.39 weeks, respectively. The FVB C3Tag parental strain fell near the median for both latency and endpoint.

Two hybrids were completely resistant to the C3Tag transgene and never developed BC tumors: BXD-BC51 and BXD-BC79. The F1s were maintained for 12 months as per study design to allow for a full year to determine if tumors arose, with palpation twice weekly to attempt to detect tumors. The 12-month endpoint was well past typical C3Tag mean latency in the parental FVB strain. GEMMs develop spontaneous tumors in response to oncogenes, and in the C3Tag with thousands of mice studied, tumor onset typically occurs from as early as 11 weeks of latency to 35 + weeks. No mammary tumors developed in BXD-BC51 and BXD-BC79 as confirmed upon dissection. Mice that did not develop a tumor by 1 year were counted as a zero in tumor traits. Of note, in addition to confirmation of accurate genotyping for the T-antigen transgene, BXD-BC79, and BXD-BC51 hybrids developed other rare tumors reported in the C3Tag parental strain such as rare mixed eccrine sweat gland tumors of the paw (Maroulakou et al. 1994). The fact that the BXD-BC F1 developed other T-antigen-related tumors (paw tumors) demonstrated correct genotyping of the GEMMs. Taken together, all mice in every strain studied developed mammary tumors except all F1 hybrids in these 2 strains. Multiplicity, represented as the total number of tumors per mouse, was quantified at the endpoint and showed significant variance across BXD-BC hybrids (Fig. 1e). The parental FVB C3Tag strain displayed a relatively low mean of 3.17 ± 0.91 tumors per mouse with a range from *N* = 1 to 7 per mouse (Fig. 1e). The BXD-BC210 F1 females developed the fewest tumors per mouse with a mean of 2.4 ± 0.68 tumors compared to the BXD-BC63 F1 which had 4-fold greater tumor multiplicity, with a mean of 8.8 ± 1.66 tumors per mouse (Fig. 1e). The average coefficient of variation (CV) was 40%, but some progeny had CVs as low as 11% (for BXD-BC1) while C3Tag parental FVB strain had the highest CV of 70% for multiplicity. The variance in CV across strains emphasizes that genetic variants exist which impact the tumor traits to induce variation beyond what is well established in the C3Tag genetically engineered mouse model (GEMM), which spurred our interest to further examine genetic modifiers.

In all hybrids that developed mammary tumors, total tumor mass was quantified by weighing all identified tumors at endpoint (Fig. 1f). Total tumor mass varied 3-fold across BXD-BC F1s ranging from about 1.1 grams per mouse in the BXD-BC66 to 3.3 g in the BXD-BC101. The FVB C3Tag parental strain had a mean total mass of 3.0 g with a range from 2.3 to 4.0 g per mouse. The average CV across all genomes was 42%, but some hybrids displayed a CV as low as 15% for BXD-BC1, while BXD-BC90 had the highest CV of 72%. Average tumor burden calculated as total tumor weight divided by total number of tumors (multiplicity) demonstrated variability across the BXD-BC strains (Supplementary Fig. 2).

Overall, tumor latency, progression multiplicity, mass, and burden demonstrated that germline variants in the BXD hybrids introduced high levels of heritable variation that modulate the onset, progression, and aggression of BC.

### Multiple tumor traits display significant heritability in BXD-BC hybrids

The BXD-BC model demonstrated large, heritable variance in multiple tumor traits as demonstrated by calculations for narrow-sense heritability (*h^2^*) and significance of strain effect. In descending order, age at endpoint (*h^2^* = 0.63), age at latency (*h^2^* = 0.52), tumor multiplicity (*h*^2^ = 0.36), and survival (endpoint minus latency, *h^2^* = 0.20) revealed robust heritability, with *P*-values in Table 1. Traits describing the first tumor (T1), including the T1 mammary gland location, T1 tumor weight, T1 tumor volume, and total tumor volume, did not display a significant strain effect (Table 1). Heritability calculations including the 2 BXD-BC hybrids that never developed tumors (BXD-BC51 and BXD-BC79) revealed that survival and age at endpoint are extremely heritable with *h^2^* = 0.97 and 0.93, respectively (Supplementary Table 5).

**Table 1.** Multiple tumor traits display significant heritability in BXD-BC hybrids.

Phenotype (trait)	*h* ^2^	*P*-value
Age at endpoint	0.633	2.94 × 10^−28^
Tumor latency	0.524	9.63 × 10^−19^
Multiplicity	0.357	1.54 × 10^−8^
Survival (time from latency to endpoint)	0.194	0.022
Endpoint 1 tumor >2 cm	0.152	0.172
Endpoint 3 tumors >1 cm	0.152	0.174
Total tumor weight	0.145	0.228
T1 tumor location	0.138	0.359
T1 tumor weight	0.100	0.755
T1 volume	0.077	0.940
Total tumor volume	0.074	0.955

Heritability (*h*^2^) and significance of strain effect (*P*) are shown for tumor traits collected for *N* = 26 BXD-BC hybrids, with an average of 8 replicates per cross. Strain effect was tested by ANOVA. Hybrids that did not develop tumors were excluded. Bold indicates significance *P* < 0.05.

### A QTL on chromosome 16 was identified for tumor multiplicity

**Fig. 2.**
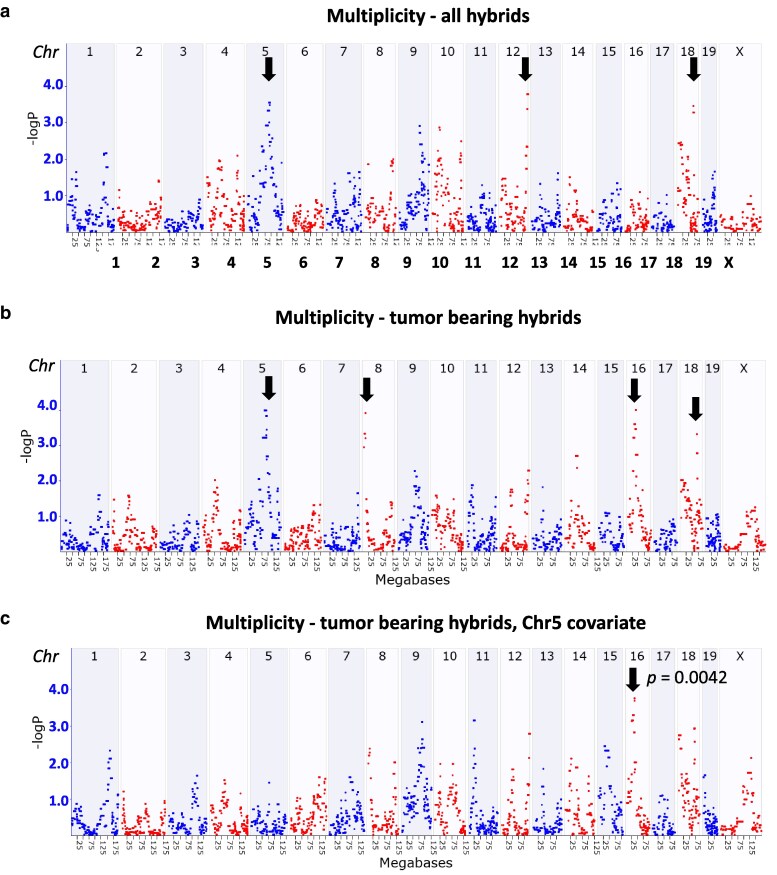
Genome-wide QTL mapping for tumor multiplicity indicated a significant QTL on chromosome 16 with candidate genes relevant in T-helper cells. a to c) Genome-wide QTL plots demonstrate the logarithm of the odds (LOD score) on the *y* axis across the genome with chromosomes noted below the *x* axis. a) Variation in tumor multiplicity maps on Chr 5 (peak at 92.8 Mb), Chr 10 (120.6 Mb), Chr 12 (116.78 Mb), and Chr 18 (68.19 Mb), with the QTL on Chr 12 having the highest LOD value (3.12, *P* = 0.0678). b). Multiplicity in only tumor-bearing hybrids was used to identify QTLs on Chr 5 (86.59 Mb), Chr 8 (10.77 Mb), Chr 16 (36.6 Mb), and Chr 18 (68.2 Mb), with the highest LOD being on Chr 16 (LOD = 3.24, *P* = 0.12). c) Chr 5 peak marker was included as a covariate revealing a significant QTL on Chr 16 (*P* = 0.004, LOD 4.0). QTLs are highlighted by black arrows.

To identify loci for tumor multiplicity, we mapped QTLs using GEMMA (Wang et al. 2003; Overall et al. 2009; Sasani et al. 2022). For tumor multiplicity, loci were identified depending upon whether all F1 types were used, including the two that never developed tumors and scoring them with zeros (GN ID BXD_ 24403, Fig. 2a), or by dropping these 2 hybrids (GN ID BXD_21528, Fig. 2b). Using all F1s, we defined 3 loci above the suggestive (−logP > 3) threshold on Chr 5 centered at 91.7 to 93.1 Mb, Chr 12 at 116.8 to 119.9 Mb, and Chr 18 at 68.2 Mb. The locus on Chr 12 had the best linkage (−logP = 3.77, Fig. 2a). However, the Chr 12 signal was not reproduced when the 2 tumor-free hybrids were excluded from the analysis. Instead, we defined 4 suggestive loci, two of which overlapped with the above, Chr 5 at 85.2 to 90.4 Mb, Chr 8 at 9.5 to 10.5 Mb, Chr 16 at 36.6 Mb, and Chr 18 at 68.2 Mb, with the highest linkage on Chr 16 (−logP = 4.0, Fig. 2b). Next, all covariate combinations were systematically tested by controlling for 1 locus at a time. Chr 5, 8, and 18 yielded no significant results in conditional analyses. When controlling for the Chr 5 QTL locus, the Chr 16 QTL locus displayed a significant LOD −logP of 4.034 (*P* = 0.0042, Fig. 2c). Therefore, the Chr 16 locus was prioritized for downstream analyses.

### Candidate genes for tumor multiplicity on chromosome 16

The QTL 1.5 LOD drop confidence interval at Chr 16: 26.4 to 44.1  Mb contained eighty annotated genes and open reading frames (ORF), 40 of which were protein-coding (Fig. 2c, Supplementary Table 6). Of these, 31 gene variants were predicted to alter protein function or splice regions in 11 genes including *Ccdc14*, *Cd86*, *Dirc2*, *Dtx3l*, *Hspbap1*, *Mylk*, *Parp14*, *Parp9*, *Sema5b*, *Slc15a2*, and *Wdr5b* (Supplementary Table 6). *Cis*-eQTLs were analyzed in several murine cell types or tissues that are specifically relevant to antitumor immunity in existing BXD family publicly available transcriptome datasets in GN: T-helper cells, T-regulatory cells, and spleen. The −logP peak score for each *cis*-eQTL and correlations between gene expression and tumor multiplicity in the indicated tissue types are reported in Table 2 (GN ID BXD_21528). Notably, the peak associated with QTL expression is at Chr 16: 36.36 Mb. Four protein-coding genes including *Slc15a2*, *Dtx3l*, *Kiaa0226 (Rubcn)*, and *Zdhhc23* have a significant *cis*-eQTL within the confidence interval in T cells and/or spleen. Solute carrier family 15 (oligopeptide transporter), member 2 (*SLC15A2*) is a protein that regulates proton-coupled amino acid transportation and is a novel biomarker in cancer (Yin et al. 2025). RUBCN is a negative regulator of autophagy (Tanaka et al. 2016). Human deltex E3 ubiquitin ligase 3L (*DTX3L*) is a ubiquitin ligase that complexes with Poly(ADP-ribose) polymerase 9 (*PARP9*) to ubiquitinate *P53* and impact DNA repair (Yan et al. 2023). Zinc finger DHHC-type palmitoyltransferase 23 (*ZDHHC23*) has protein-cysteine S-palmitoyltransferase activity which is essential to reprogram lipogenesis and maintain homeostasis in T-cell immune responses (Jeong et al. 2023). Among these, *Slc15a2* and *Dtx3l* revealed high −logP scores, particularly in T-regulatory cells (18.52 and 12.19, respectively) and T-helper cells (13.78 and 5.42, respectively) with positive correlation to tumor multiplicity. In the spleen, *Kiaa0226 (Rubcn)* demonstrated a high −logP score of 12.48 with a significant positive correlation with tumor multiplicity (rho = 0.54, *P* = 0.017). *Dtx3l* exhibited a significant positive correlation (rho = 0.9, *P* = 0.037) with multiplicity in T-helper cells. Conversely, *Zdhhc23*, with a −logP score of 7.26 in T-helper cells, displayed a significant negative correlation with tumor multiplicity (rho = −0.90, *P* = 0.037). Taken together, Chr 16 QTL analysis identified 80 candidates narrowed down to 11 genes of interest, with *cis*-eQTL mapping in immune-relevant tissues highlighting positive (*Slc15a2*, *Dtx3l*, and *Rubcn*) or negative (*Zdhhc23*) drivers of tumor multiplicity.

**Table 2.** Correlation of chromosome 16 *cis*-eQTL genes associated with various tissues and tumor multiplicity.

Cis-eQTL genes in Chr 16 −logP Peak position						Tumor multiplicity_strains with only tumor (GeneNetword ID: BXD_21528)			
Chr	Gene	Tissue	Probe ID	−logP	Peak Position	*R*	*r P*-value	Rho	Rho *P*-value
16	*Slc15a2*	T-helper cells	1417600_at	13.78	Chr 16: 32.540701	0.48	0.409	0.60	0.285
16	*Slc15a2*	T-regulatory cells	1417600_at	18.52	Chr 16: 32.540701	0.52	0.373	0.60	0.285
16	*Dtx3l*	T-helper cells	1439825_at	5.42	Chr 16: 34.308361	0.64	0.241	0.90	0.037
16	*Dtx3l*	T-regulatory cells	1439825_at	12.19	Chr 16: 34.308361	0.71	0.183	0.90	0.037
16	*Kiaa0226*	Spleen	10439092	12.48	Chr 16: 36.361531	0.52	0.024	0.54	0.017
16	*Zdhhc23*	T-helper cells	1441069_at	7.26	Chr 16: 36.946692	−0.87	0.053	−0.87	0.037

A subset of genes from the Chr 16 QTL based on proximity to within ±5 Mb of each other is shown. A score of −logP and peak position were generated in GEMMA for Chr 16 QTL, and *r* Pearson's or rho Spearman's correlation values were reported from different tissues.

### Candidate genes for tumor latency on chromosome 10

**Fig. 3.**
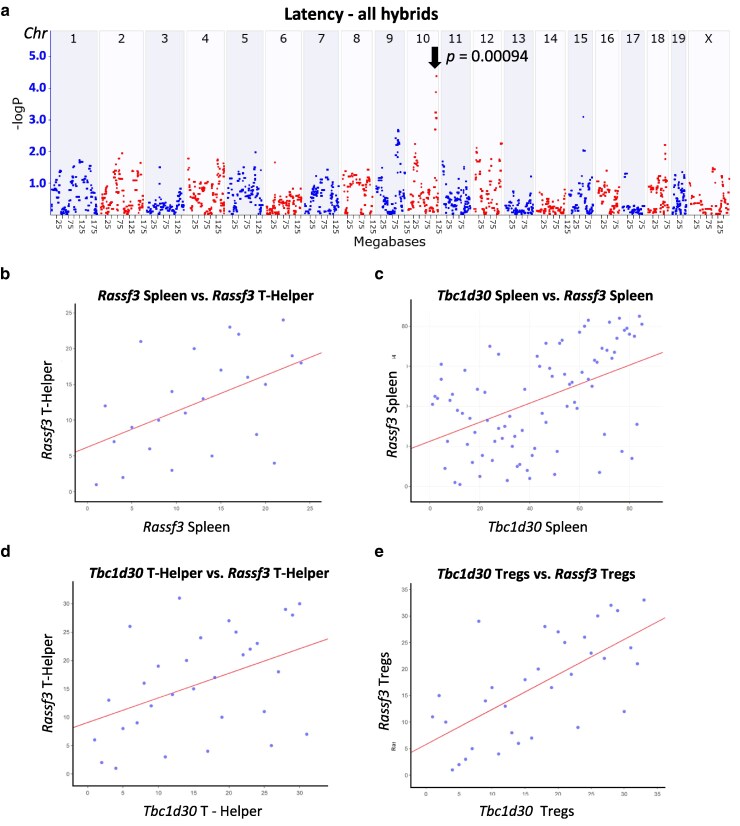
A significant QTL for tumor latency was identified on chromosome 10. a). QTLs associated with tumor latency (age in weeks) were examined in GEMMA. A QTL is noted with a arrow at 120.6 Mb on Chr 10 (*P* = 9.4 × 10^−5^). b to e) Scatter plots of correlations between *Rassf3* and *Tbc1d30* in various tissues. Each data point represents the mean gene expression in the noted murine tissue or cell types from a publicly available dataset in GeneNetwork. Data for each mouse is shown as a dot for gene expression, and the correlation line is drawn. b) *Rassf3* expression in the spleen (GN ID 10372844, *x* axis) is positively correlated with *Rassf3* in T-helper cells (GN ID 1448547_at, rho = 0.50, *P* = 1.3 × 10^−2^, *n* = 24. c) *Tbc1d30* expression in the spleen (GN ID 10372831, *x* axis) is positively correlated with *Rassf3* expression in the spleen (GN ID 10372844, *y* axis, rho = 0.48, *P* = 4.3 × 10^−6^, *n* = 85). d) *Tbc1d30* (GN ID 1430607_at, *x* axis) and *Rassf3* (GN ID 1448547_at, *y* axis) are positively correlated in T-helper cells. Rho = 0.434, *P* = 1.48 × 10^−2^, *n* = 31. e) *Tbc1d30* (GN ID 1430607_at, *x* axis) and *Rassf3* (GN ID 1448547_at, *y* axis) are positively correlated in T-regulatory (Treg) cells. Rho = 0.660, *P* = 2.934 × 10^−6^, *n* = 33.

We next examined tumor latency (GN ID BXD_24402) wherein resistant hybrids that failed to develop a tumor were included in QTL analysis. A significant locus for tumor latency on Chr 10 was identified with a peak at 120.6 Mb (*P* = 9.4 × 10^−5^, Fig. 3a). The confidence interval extended from 117.3 to 121.5 Mb and included 121 annotated genes, of which 27 were protein-coding (Supplementary Table 7). The eQTL analysis revealed robust gene expression in tissues relevant to antitumor immunity, such as T-helper cells, T-regulatory cells, and spleen (Table 3). Of the 6 protein-coding candidate genes in the Chr 10 QTL, 3 genes were identified with a significant *cis*-eQTL in at least one of the relevant tissues: *Cand1*, *Tbc1d30*, and *Rassf3*. A correlation matrix of the Chr 10 QTL and eQTL genes identified 5 candidates including *Cand1*, *Lemd3*, *Tbc1d30*, *Gns*, and *Rassf3*, revealed strong correlations with each other in at least one of the examined tissues (*data not shown*). Among these, 3 genes exhibited significant *cis*-eQTL in the respective antitumor-related tissues: *Gns* (T-helper cells −logP 5.0), *Tbc1d30* (T-regulatory cells −logP 8.65; spleen −logP 9.19), and *Rassf3* (spleen −logP 7.2). *GNS* is a lysosomal enzyme that impacts cancer cell proliferation (Trybus et al. 2023). TBC1 domain family member 30 (*TBC1D30*, also known as KIAA0984) is a RAB-GTPase-activating protein (Nottingham and Pfeffer 2015). The Ras association domain family member 3 (*RASSF3*) gene is a member of the subfamily of *RAS* effectors with inactivation of RASSF family genes in many human tumors (Bovo et al. 2020). *GNS* and *TBC1D30* have predicted impacts on prostate cancer and BC (Mastrangelo et al. 2019; Cheng et al. 2021). Analysis demonstrated that *Tbc1d30* and *Rassf3* expression in spleen and T-regulatory cells showed positive correlations with tumor latency (Table 3). Interestingly, a positive correlation was evident between *Rassf3* expression in spleen and T-helper cells (rho = 0.5, *P* = 0.013, Fig. 3b). *Rassf3* and *Tbc1d30* also have a positive correlation to each other in the spleen (rho = 0.47, *P* = 4.3 × 10^−6^), T-helper cells (rho = 0.434, *P* = 0.015), and T-regulatory cells (rho = 0.660, *P* = 2.934 × 10^−5^, Fig. 3c to e). Taken together, the correlation between *Tbc1d30* and *Rassf3* suggests a potential *cis*-regulatory role across immune cell types, indicating their possible involvement in shared biological processes. No significant correlation was found with *Gns* (data not shown) in either spleen, T-helper cells, or T-regulatory cells. Overall, a QTL for tumor latency on Chr 10 identified *Tbc1d30* and *Rassf3* as key candidate genes with significant *cis*-eQTLs in immune-related tissues, showing strong positive correlations with each other and with tumor latency, suggesting a shared *cis*-regulatory role in antitumor immunity.

**Table 3.** Correlation of chromosome 10 *cis*-eQTL genes associated with Various tissues and tumor latency.

*Cis*-eQTL genes in Chr 10 −logP Peak position						Tumor latency_strains including without tumor (GeneNetword ID: BXD_24402)			
Chr	Gene	Tissue	Probe ID	−logP	Peak Position	*R*	*r P*-value	Rho	Rho *P*-value
10	*Gns*	T-helper cells	1433488_x_at	4.97	Chr 10: 120.212361	−0.588	0.220	−1.000	1.50 × 10^−40^
10	*Rassf3*	Spleen	10372844	7.18	Chr 10: 120.634059	0.559	0.016	0.278	0.264
10	*Tbc1d30*	Spleen	10372831	9.19	Chr 10: 120.634059	0.474	0.047	0.359	0.143
10	*Msrb3*	T-regulatory cells	1439151_at	4.07	Chr 10: 120.783223	0.729	0.100	0.829	0.042
10	*Tbc1d30*	T-regulatory cells	1430607_at	8.65	Chr 10: 121.266431	0.665	0.150	0.886	0.019
10	*Rassf3*	T-regulatory cells	1448547_at	6.52	Chr 10: 121.411254	0.782	0.066	0.551	0.257

A subset of genes from the Chr 10 QTL based on proximity to within ±5 Mb of each other is shown. A score of -logP and peak position were generated in GEMMA for Chr 10 QTL, and *r* Pearson's or rho Spearman's correlation values were reported from different tissues.

### Histological analysis of tumors revealed variability in cancer traits, but heritability was generally low

Mitosis per high-powered field varied from 10 to 80 mitotic nuclei per high-powered field (Supplementary Fig. 3a). EMT was variable across hybrids with means ranging from 1 to 2.7 in each F1 (Supplementary Fig. 3b). Vascularity and stromal content was only moderately variable across hybrids although most tumors displayed a score of 2 on average (Supplementary Fig. 3c). Likewise, tumor necrosis was also moderately variable across hybrids with a mean of 2.4 (Supplementary Fig. 3d). Heritability was calculated for each histologic phenotype quantified. The highest heritability was for mitosis per high-powered field and vascularity and stroma with an *h^2^* of 0.34 and 0.33, respectively; however, analysis did not reveal any significant strain effect for histologic traits (Supplementary Table 8).

### Gene variants and eQTL analysis identified 2 significant QTL associated with histologic traits

The necrosis score (GN BXD_27515) revealed a significant QTL on Chr 4 with a LOD score of 5.86 and a peak position at Chr 4 105.2 Mb, and a QTL interval at 103.64 to 105.24 Mb (Supplementary Fig. 4a). Further focusing on the 1.5 LOD drop confidence QTL interval, 3,219 gene variants were identified including 23 annotated genes and 6 of which are protein-coding genes including *Dab1*, *C8b*, *C8a*, *Fyb2*, *Plpp3*, and *Prkaa2* (Supplementary Fig. 4b and Supplementary Table 9). The stroma and vascularity score (GN BXD_27514) identified a locus on Chr 12 with a LOD of 5.12 and a peak at 67 to 68.6 Mb and a confidence interval 64.4 and 69.6  Mb, containing 3,752 sequence variants and 172 annotated genes, 43 of which are protein-coding genes (Supplementary Fig. 4d and Supplementary Table 10). Notably, no significant *cis*-eQTL gene variants were identified in either region under the conditions analyzed.

### Joint mouse–human phenome-wide association study (PheWAS) analysis revealed syntenic regions of interest

The translational validity of the candidate genes was tested based on PheWAS studies in humans including diseases such as BC. The loci on Chr 16 and Chr 10 identified in the BXD-BC model are syntenic to human Chr 3 and Chr 12 regions, respectively (Table 4). Several PheWAS databases (Supplementary Table 2) were evaluated to identify single-nucleotide variants (SNV) associated with cancer phenotypes and their closest genes. The type of variants was included based on predicted functional consequences, and missense variants with a significant *P*-value < 0.05 (Table 5). Gene variants without any association with cancer-related phenotypes were ignored.

**Table 4.** Cross-species human phenome-wide association study (PheWAS) identified genes and regions of interest.

QTL	Mouse proximal gene	Mouse distal gene	Mouse proximal gene location	Mouse distal gene location	Approx. mouse region length	Human proximal gene location	Human distal gene location	Approx. human region length
Chr 10	*Cpsf6*	*Rassf3*	Chr 10: 117.4	Chr 10: 121.4	4.05	Chr 12: 69.3	Chr 12: 64.6	—
Chr 16	*Heg1*	*Golgb1*	Chr 16: 33.7	Chr 16: 36.9	3.20	Chr 3: 125.1	Chr 3: 121.7	—

The human genomic regions syntenic to the significant mouse QTL regions on chromosome 10 for tumor latency and chromosome 16 for tumor multiplicity were identified. For each QTL, the most proximal and distal genes were identified, and the location of these genes in mouse and human was noted.

**Table 5.** Gene variants on huChr 12 and huChr 3 and PheWas data associated with cancer and cancer-related phenotypes.

Index	Gene symbol	Variant type	Variant ID	Gene variant location (Chr: Mb)	Library	PheWAS	*P*-value
1	CPSF6	Intron variant (A > G)	rs554429384	12: 69573035	https://pheweb.jp/	Ovarian cancer	2.80 × 10^−5^
2	CPM	Intron variant (C > T)	rs9943849	12:67657023	https://phewascatalog.org/	Malignant neoplasm of the renal pelvis	3.50 × 10^−2^
		Intron variant (C > T)	rs9943849	12 67657023		Benign neoplasm of the brain and other parts of the nervous system	4.17 × 10^−2^
		Intron variant (G > A)	rs7304105	12:69181478	https://pheweb.jp/	Breast cancer	4.20 × 10^−10^
		3′ UTR (A > G)	rs557499913	12:68841838	http://pheweb.sph.umich.edu/	Benign neoplasm of the uterus	7.90 × 10^−9^
		3′ UTR (A > G)	rs557499913	12:68841838		Uterine leiomyoma	3.40 × 10^−9^
4	MDM2	Intron variant (G > A)	rs7304105	12:69181478	https://pheweb.jp/	Breast cancer	4.20 × 10^−10^
5	SLC35E3	Intron variant (G > A)	rs7304105	12:69181478	https://pheweb.jp/	Breast cancer	4.20 × 10^−10^
6	NUP107	Intron variant (G > A)	rs7304105	12:69181478	https://pheweb.jp/	Breast cancer	4.20 × 10^−10^
		Missense (G > A)	rs139991199	12:69083373	https://biobankengine.stanford.edu/	Age at cancer diagnosis	9.48 × 10^−6^
		Intron variant (C > T)	rs551692899	12:68646350	http://pheweb.sph.umich.edu/	Benign neoplasm of the uterus and uterine leiomyoma	3.40 × 10^−9^
		3′ UTR (A > G)	rs557499913	12:68841838		Uterine leiomyoma	3.40 × 10^−9^
		3′ UTR (A > G)	rs557499913	12:68841838		Benign neoplasm of the uterus	7.90 × 10^−9^
		Intron variant (A > G)	rs183391298	12:68683185		Benign neoplasm of the colon	2.20 × 10^−7^
7	RAP1B	Regulatory region variant (C > G)	rs190966000	12:68565994	http://pheweb.sph.umich.edu/	Benign neoplasm of the colon	1.50 × 10^−8^
		Intron variant (C > T)	rs551692899	12:68646350		Benign neoplasm of the uterus	1.60 × 10^−7^
		Intron variant (A > G)	rs2546509	12:69083237	https://pheweb.jp/	Breast cancer	5.20 × 10^−9^
8	GRIP1	Intron variant (CTGTG > C)	rs916631817	12:66563178	http://pheweb.sph.umich.edu/	Benign neoplasm of the uterus	3.40 × 10^−7^
9	IL22	Splice donor (A > G)	rs867810424	12:68647041	https://biobankengine.stanford.edu/	Breast fibroadenoma	3.06 × 10^−6^
10	IRAK3	Missense (A > G)	rs139342884	12:66610970	https://biobankengine.stanford.edu/	Congenital malformations of the breast	5.49 × 10^−4^
		Intergenic variant (T > C)	rs147746198	12:66097705	http://pheweb.sph.umich.edu/	Hyperplasia of the prostate	9.10 × 10^−7^
		Intergenic variant (C > A)	rs75184621	12:66179444		Hyperplasia of the prostate	9.10 × 10^−7^
11	TMBIM4	Missense (G >A)	rs200879651	12:66563658	https://biobankengine.stanford.edu/	Congenital malformations of the breast	5.18 × 10^−13^
		Intron variant (A > C)	rs117368235	12:66543913		Congenital malformations of the breast	1.15 × 10^−5^
		Intergenic variant (T > C)	rs147746198	12:66097705	http://pheweb.sph.umich.edu/	Hyperplasia of the prostate	9.10 × 10^−7^
		Intron variant (C > A)	rs34750837	12:66161838		Hyperplasia of the prostate	9.10 × 10^−7^
12	LLPH	Intergenic variant (T > C)	rs147746198	12:66097705	http://pheweb.sph.umich.edu/	Hyperplasia of the prostate	9.10 × 10^−7^
		Intron variant (C > A)	rs34750837	12:66161838		Hyperplasia of the prostate	9.10 × 10^−7^
13	HMGA2	Intergenic variant (T > C)	rs12424086	12:64650776	https://phewascatalog.org/	Cancer of other female genital organs	4.60 × 10^−3^
		Intergenic variant (T > C)	rs12424086	12:64650776		Ovarian cancer	9.35 × 10^−3^
		3′ UTR (C > A/C > G/C > T)	rs8756	12:64646019		Uterine cancer	9.64 × 10^−3^
		Intron variant (C > A/C > T)	rs7979673	12:64513524		Uterine cancer	1.13 × 10^−2^
		Intergenic variant (T > C)	rs12424086	12:64650776		Hepatic cancer, primary	2.24 × 10^−2^
		Intergenic variant (T > C)	rs12424086	12:64650776		Pancreatic cancer	2.29 × 10^−2^
		3′ UTR (C > A/C > G/C > T)	rs8756	12 64646019		Benign neoplasm of other parts of the digestive system	6.67 × 10^−3^
		3′ UTR (C > A/C > G/C > T)	rs8756	12:64646019		Benign neoplasm of other endocrine glands	2.33 × 10^−2^
		3′ UTR (C > A/C > G/C > T)	rs8756	12:64646019		Skin neoplasm of uncertain behavior	3.99 × 10^−2^
14	MSRB3	Intron variant (T > A/T > C/T > G)	rs10506525	12:64069645	https://phewascatalog.org/	Pancreatic cancer	3.33 × 10^−2^
		Intron variant (T > A/T > C/T > G)	rs10506525	12:64069646		Benign neoplasm of respiratory and intrathoracic organs	4.25 × 10^−2^
		Intron variant (C > G)	rs117523829	12:65707367		Congenital malformations of the breast	4.11 × 10^−7^
15	TBC1D30	Intron variant (A > G)	rs939876	12 63538385	https://phewascatalog.org/	Mouth cancer	1.47 × 10^−2^
		Intron variant (A > G)	rs939876	12 63538385		Neoplasm of uncertain behavior	6.08 × 10^−3^
		Intron variant (A > G)	rs939876	12 63538385		Hx of malignant neoplasm of the oral cavity and pharynx	2.43 × 10^−2^
		Intron variant (A > G)	rs939876	12 63538385		Malignant neoplasm of the brain and nervous system	3.27 × 10^−2^
		Intron variant (A > G)	rs11175566	12:65214248	https://biobankengine.stanford.edu/	Congenital malformations of the breast	7.76 × 10^−6^
		3′ UTR (C > G)	rs117754169	12:65272906		Tongue cancer	4.02 × 10^−5^
		Intron variant (C > T)	rs7313651	12:64618224	https://pheweb.jp/	Prostate cancer	1.80 × 10^−17^
		Intron variant (A > G)	rs1147107	12:64632562		Prostate cancer	1.80 × 10^−17^
		5′ UTR (A > G)	rs1245183	12:65059192		Prostate cancer	1.80 × 10^−17^
16	GNS	Intron variant (T > C)	rs7968403	12:65012824	https://pheweb.jp/	Prostate cancer	1.20 × 10^−19^
		Intron variant (A > G)	rs1147107	12:65026342		Prostate cancer	1.20 × 10^−19^
		Intron variant (T > G)	rs1244869	12:6507533		Prostate cancer	1.20 × 10^−19^
		Intron variant (T > C)	rs7968403	12:65012824		Breast and prostate cancer	4.30 × 10^−6^
		Intron variant (A > G)	rs1147107	12:65026342		Breast and prostate cancer	4.30 × 10^−6^
		Intron variant (T > G)	rs1244869	12:6507533		Breast and prostate cancer	4.30 × 10^−6^
		5′ UTR (A > G)	rs1245183	12:65059192	https://pheweb.jp/	Prostate cancer	1.80 × 10^−17^
17	RASSF3	Intron variant (A > G)	rs1245183	12:65059192	https://pheweb.jp/	Prostate cancer	1.80 × 10^−17^
		Intron variant (T > C)	rs7968403	12:65012824		Prostate cancer	1.20 × 10^−19^
		Intron variant (T > C)	rs7968403	12:65012824		Breast and prostate cancer	4.30 × 10^−6^
		Intron variant (T > C)	rs7968403	12:65012824		Esophageal cancer	3.30 × 10^−3^
		Intron variant (A > G)	rs1147107	12:65026342	http://pheweb.sph.umich.edu/	Breast and prostate cancer	4.30 × 10^−6^
		Intron variant (T > G)	rs1244869	12:6507533		Breast and prostate cancer	4.30 × 10^−6^
		Intron variant (C > T)	rs7313651	12:64618224		Breast cancer	5.10 × 10^−2^
		Intron variant (C > T)	rs7313651	12:64618224		Breast cancer (female)	6.00 × 10^−2^
		Intron variant (C > T)	rs7313651	12:64618224		Prostate cancer	3.90 × 10^−3^
		Intron variant (A > G)	rs1147107	12:64632562		Prostate cancer	7.90 × 10^−3^
		Intron variant (A > G)	rs1147107	12:64632562		Cancer of intrathoracic organs	1.90 × 10^−2^
		Intron variant (A > G)	rs1147107	12:64632562		Pancreatic cancer	2.90 × 10^−2^
		Intron variant (A > G)	rs1147107	12:64632562		Breast cancer (female)	8.40 × 10^−2^
		Intron variant (A > G)	rs1147107	12:64632562		Breast cancer	9.00 × 10^−2^
		Intron variant (G > T)	rs2682728	12:64682718		Malignant neoplasm of the uterus	1.30 × 10^−2^
		Intron variant (G > T)	rs2682728	12:64682718		Cancer of intrathoracic organs	2.40 × 10^−2^
		Intron variant (T > G)	rs1244869	12:64681552		Pancreatic cancer	1.80 × 10^−2^
18	HEG1	Intron variant (C > A/C > G)	rs190282002	3:124771081	https://pheweb.jp/	Pharyngeal and laryngeal cancer	1.70 × 10^−5^
19	MUC13	Intron variant (C > A/C > G)	rs190282002	3:124771081	https://pheweb.jp/	Pharyngeal and laryngeal cancer	1.70 × 10^−5^
20	ITGB5	Missense (C > T)	rs61736434)	3:124515354	https://biobankengine.stanford.edu/	Congenital malformations of the breast	2.33 × 10^−10^
		Intron variant (A > G)	rs760416624	3:124714206	http://pheweb.sph.umich.edu/	Benign neoplasm of the colon	6.70 × 10^−7^
21	UMPS	Intron variant (A > G)	rs760416624	3:124714206	http://pheweb.sph.umich.edu/	Benign neoplasm of the colon	6.70 × 10^−7^
22	KALRN	Intron variant (C > T)	rs762914024	3:124457563	http://pheweb.sph.umich.edu/	Benign neoplasm of the colon	6.70 × 10^−7^
23	MYLK	Intron variant (A > C)	rs76810901	3:123380319	https://biobankengine.stanford.edu/	Breast cancer	3.53 × 10^−5^
		Intron variant (C > A)	rs9851418	3:123435754		Benign breast lump	2.47 × 10^−5^
		Intron variant (C > T)	rs116256520	3:123356070		Cervical intraepithelial neoplasia/precancerous cells of the cervix	2.60 × 10^−5^
24	PDIA5	Missense (A > G)	rs368837820	3:122880168	https://biobankengine.stanford.edu/	Benign neuroma	1.77 × 10^−9^
		Missense (G > A)	rs202014966	3:122942523		Congenital malformations of the breast	3.80 × 10^−10^
25	PARP15	Missense (A > G)	rs753177428	3:122635033	http://pheweb.sph.umich.edu/	Malignant neoplasm, other	3.10 × 10^−6^
26	DTX3L	Missense (A > G)	rs753177428	3:122635033	http://pheweb.sph.umich.edu/	Malignant neoplasm, other	3.10 × 10^−6^
27	PARP9	Missense (A > G)	rs753177428	3:122635033	http://pheweb.sph.umich.edu/	Malignant neoplasm, other	3.10 × 10^−6^
28	KPNA1	3′ UTR (C > T)	rs189676830	3:122424634	http://pheweb.sph.umich.edu/	Bladder cancer	7.50 × 10^−8^
		Intron variant (C > T)	rs572858475	3:122389010		Bladder cancer	7.50 × 10^−8^
		3′ UTR (C > T)	rs189676830	3:122424634		Malignant neoplasm of the bladder	4.40 × 10^−7^
		Intron variant (C > T)	rs572858475	3:122389010		Malignant neoplasm of the bladder	4.40 × 10^−7^
29	WDR5B	3′ UTR (C > T)	rs189676830	3:122424634	http://pheweb.sph.umich.edu/	Bladder cancer	7.50 × 10^−8^
		Intron variant (C > T)	rs572858475	3:122389010		Bladder cancer	7.50 × 10^−8^
		3′ UTR (C > T)	rs189676830	3:122424634		Malignant neoplasm of the bladder	4.40 × 10^−7^
		Intron variant (C > T)	rs572858475	3:122389010		Malignant neoplasm of the bladder	4.40 × 10^−7^
30	FAM162A	3′ UTR (C > T)	rs189676830	3:122424634	http://pheweb.sph.umich.edu/	Bladder cancer	7.50 × 10^−8^
		Intron variant (C > T)	rs572858475	3:122389010		Bladder cancer	7.50 × 10^−8^
		3′ UTR (C > T)	rs189676830	3:122424634		Malignant neoplasm of the bladder	4.40 × 10^−7^
		Intron variant (C > T)	rs572858475	3:122389010		Malignant neoplasm of the bladder	4.40 × 10^−7^
31	CCDC58	3′ UTR (C > T)	rs189676830	3:122424634	http://pheweb.sph.umich.edu/	Bladder cancer	7.50 × 10^−8^
		Intron variant (C > T)	rs572858475	3:122389010		Bladder cancer	7.50 × 10^−8^
		3′ UTR (C > T)	rs189676830	3:122424634		Malignant neoplasm of the bladder	4.40 × 10^−7^
		Intron variant (C > T)	rs572858475	3:122389010		Malignant neoplasm of the bladder	4.40 × 10^−7^
32	CSTA	3′ UTR (C > T)	rs189676830	3:122424634	http://pheweb.sph.umich.edu/	Bladder cancer	7.50 × 10^−8^
		Intron variant (C > T)	rs572858475	3:122389010		Bladder cancer	7.50 × 10^−8^
		3′ UTR (C > T)	rs189676830	3:122424634		Malignant neoplasm of the bladder	4.40 × 10^−7^
		Intron variant (C > T)	rs572858475	3:122389010		Malignant neoplasm of the bladder	4.40 × 10^−7^
33	CASR	Missense (G > A/G > T)	rs1801725	3:123486447	https://phewascatalog.org/	Colon cancer	1.98 × 10^−2^
		Missense (G > A/G > T)	rs1801725	3:123486447		Kidney cancer and urinary organs	2.16 × 10^−2^
		Missense (G > A/G > T)	rs1801725	3:123486447		Bladder cancer and neoplasms	2.51 × 10^−2^
		Missense (G > A/G > T)	rs1801725	3:123486447		Cancer of other male genital organs	2.82 × 10^−2^
		Missense (G > A/G > T)	rs1801725	3:123486447		Bladder cancer and neoplasms	2.51 × 10^−2^
		Missense (G > A/G > T)	rs1801725	3:123486447		Malignant neoplasm of the kidney and other urinary organs	3.88 × 10^−2^
		Downstream Gene variant (G > T)	rs879868803	3:122291773	http://pheweb.sph.umich.edu/	Bladder cancer	9.70 × 10^−8^
		Intron variant (C > T)	rs572858475	3:122389010		Malignant neoplasm of the bladder	5.40 × 10^−7^
34	CD86	Intron variant (G > A)	rs9282641	3:123279458	https://phewascatalog.org/	Uterine cancer	4.72 × 10^−2^
		Intron variant (G > A)	rs9282641	3:123279458		Malignant neoplasm of the renal pelvis	7.15 × 10^−3^
		Intron variant (G > A)	rs9282641	3:123279458		Benign neoplasm of the thyroid glands	7.84 × 10^−3^
		Intron variant (G > A)	rs9282641	3:123279458		Malignant neoplasm of the brain and nervous system	4.89 × 10^−2^
		Intergenic variant (C > T)	rs1225678357	3:121969387	http://pheweb.sph.umich.edu/	inflammatory diseases in the breast	9.20 × 10^−7^
35	SLC15A2	3′ UTR (A > C/A > G)	rs4285028	3:123143354	https://phewascatalog.org/	Neoplasm of unspecified nature of the digestive system	2.31 × 10^−2^
		3′ UTR (A > C/A > G)	rs4285028	3:123143354		Other benign neoplasms of connective and other soft tissue	4.12 × 10^−2^
		3′ UTR (A > C/A > G)	rs4285028	3:123143354		Hepatic cancer, primary	3.32 × 10^−2^
		3′ UTR (A > C/A > G)	rs4285028	3:123143354		Other congenital anomalies of the skin	1.36 × 10^−2^
		3′ UTR (A > C/A > G)	rs4285028	3:123143354		Congenital anomalies of the urinary system	2.89 × 10^−2^
		3′ UTR (A > C/A > G)	rs4285028	3:123143354		Genitourinary congenital anomalies	3.23 × 10^−2^
		3′ UTR (A > C/A > G)	rs4285028	3:123143354		Myeloid leukemia	4.19 × 10^−^ × 10^−^
		Intron variant (C > G)	rs551291937	3:121860679	http://pheweb.sph.umich.edu/	Non-Hodgkins lymphoma	1.70 × 10^−7^
		Intron variant (C > G)	rs551291937	3:121860679		Cancer of other lymphoid, histiocytic tissue	2.80 × 10^−7^
		Splice Donor (T > C)	rs142221393	3:121641710	https://biobankengine.stanford.edu/	Appendix cancer	3.19 × 10^−9^
		Intergenic variant (C > T)	rs1225678357	3:121969387	http://pheweb.sph.umich.edu/	Inflammatory diseases in the breast	9.20 × 10^−7^
36	EAF2	Intron variant (C > G)	rs551291937	3:121860679	http://pheweb.sph.umich.edu/	Non-Hodgkins lymphoma	1.70 × 10^−7^
		Intron variant (C > G)	rs551291937	3:121860679		Cancer of other lymphoid, histiocytic tissue	2.80 × 10^−7^
		Intergenic variant (C > T)	rs1225678357	3:121969387		Inflammatory diseases in the breast	9.20 × 10^−7^
37	IQCB1	Intron variant (C > G)	rs551291937	3:121860679	http://pheweb.sph.umich.edu/	Non-Hodgkins lymphoma	1.70 × 10^−7^
		Intron variant (C > G)	rs551291937	3:121860679		Cancer of other lymphoid, histiocytic tissue	2.80 × 10^−7^
		Intergenic variant (C > T)	rs1225678357	3:121969387		Inflammatory diseases in the breast	9.20 × 10^−7^

Annotations of syntenic mouse genes to human protein-coding genes include gene symbol, variant type, variant ID, gene variant location (Chr: Mb), PheWas reported, and phenotype *P*-value.

From the genes identified in the Chr 16 QTL associated with tumor multiplicity, PheWAS analysis in the UK Biobank and Biobank Japan databases did not identify specific significant gene variants per se that associated with a specific BC phenotype (Canela-Xandri et al. 2018; Sakaue et al. 2021). However, an interesting intergenic variant located between *SLC15A2* and *ILDR1* (C > T, rs1225678357) was found to alter the function of 5 genes (*KPNA1*, *CD86*, *ILDR1*, *SLC15A2*, and *EAF2*) in “inflammatory disease of the breast” (*P* = 9.2 × 10^−7^). Similarly, PheWAS analysis identified variants associated with other cancers. Two gene variants that co-regulated bladder cancer phenotypes in humans were identified. A *KPNA1* missense variant (C > T, rs189676830) located at the 3′ UTR region at Chr 3 122.424 Mb, and *FAM162A* intron variant (C > T, rs572858475) at Chr 3:122.389 Mb impacted 6 proximal genes (*KPNA1*, *WRD5B*, *FAM162A*, *CCDC58*, *CSTA*, and *CASR*), which associated with “malignant neoplasm of bladder” (*P* = 4.40 × 10^−7^) and “bladder cancer” (*P* = 7.5 × 10^−8^). Interestingly, a poly(ADP-ribose) polymerase family member 15 (*PARP15*) missense variant (A > G, rs753177428) with altered protein structure is associated with a “malignant neoplasm of an unknown kind” (*P* = 3.10 × 10^−6^) and influences *DTX3L* and a nearby gene critical to DNA repair, *PARP9* (Supplementary Fig. 5a). Additionally, 2 genes *IQCB1* and *SLC15A2* identified through *cis*-eQTL analysis were found to be affected by an *IAF2* intron variant (C > G, rs551291937) involved in “non-Hodgkins lymphoma” (*P* = 1.70 × 10^−7^) and “cancer of other lymphoid, histiocytic tissue” (*P* = 2.80 × 10^−7^).

The 3 genes identified in the Chr 10 QTL associated with BXD-BC tumor latency found on human Chr 12 (*TBC1D30*, *GNS*, and *RASSF3*) displayed significant associations with human prostate cancer and BC (Table 5). Notably, the *RASSF3* intron missense variant (T > C, rs7968403) exhibited significant associations with cancers of the prostate (*P* = 1.2 × 10^−19^) and breast (*P* = 4.3 × 10^−6^). Additionally, 2 other missense variants (C > T, rs7313651, and A > GT > C, rs1245183) within the *RASSF3* intron region were linked to “breast cancer” (*P* = 5.10 × 10^−5^) and “prostate cancer” (*P* = 1.8 × 10^−17^), which impacted the expression of nearby *GNS* and *TBC1D30* genes, respectively (Supplementary Fig. 5b). Furthermore, 2 additional *SLC35E3* intron variants (G > A, rs7304105 and C > T, rs74705899) exhibited significant associations with “breast cancer” (*P* = 4.2 × 10^−10^), affecting *CPM*, *MDM2*, and *NUP107* gene expression. Interestingly, variants noted in *SLC35E3*, which are associated with BC and impacted *MDM2* and *NUP107* gene expression, were of specific interest because of recent work using transcriptome-wide association study (TWAS) analysis in African American females (Li et al. 2024; Ping et al. 2024). Lastly, an intragenic variant at human Chr 12:66, located between *HMGA2* and *LLPH* (T > C, rs147746198), was found to alter gene expression of *IAK3*, *TMBIM4*, and *LLPH* in prostate hyperplasia (*P* = 9.10 × 10^−7^, Table 5). In sum, conserved genes were identified using the BXD-BC preclinical model that aligned with gene variants that impact BC and other cancer or cancer-associated phenotypes.

### Candidate analysis in patients demonstrated combined GNS, RASSF3, and TBC1D30 expression associated with significantly reduced breast cancer patient survival

**Fig. 4.**
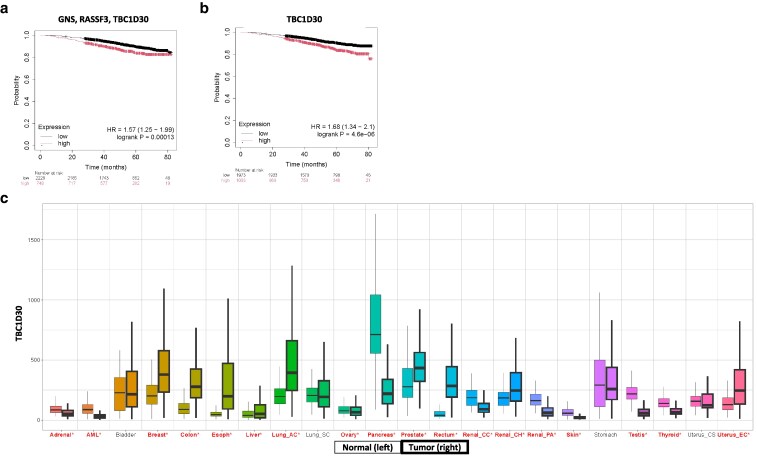
GNS, RASSF3, and TBC1D30 are associated with poor BC patient overall survival. a and b) Overall survival probability in BC patients is graphed for tumors with expression of *GNS*, *RASSF3*, and *TBC1D30* combined (a) or only for *TBC1D30* (b) with high expression compared to low expression using the KmPlotter webserver and breast RNA-seq data. Hazard ratio (HR) and log-rank *P*-values are 1.49 and *P* = 0.00071 (a) and 1.68 and *P* = 0.0000046 (b). A total of *N* = 2,976 breast cancer patients are included for a and b. c) Differential RNA-seq gene expression pan-cancer analysis on the TNMplot webserver in normal, tumor, or metastatic samples from patients indicates normal (left, thin outline) or tumor (right, thick outline) gene expression with asterisk indicating significance by Mann–Whitney analysis (*P < 0.01). Patient sample data include normal (15,648), tumor (40,442), and metastatic (848) tissue. Tumor types are shown for AML (acute myeloid leukemia), Esoph (esophageal), AC (adenocarcinoma), SC (squamous carcinoma), CC (cell carcinoma), CH (chromophobe), PA (papillary), CS (carcinosarcoma), and EC (endometrial cancer).

Analysis of GNS, RASSF3, and TBC1D30 in the RNA-seq database using the web-based analysis tool KMplotter (Gyorffy 2024a,b) demonstrated that greater expression of the 3 genes combined (Fig. 4a) and notably TBC1D30 (Fig. 4b) revealed significantly poorer OS in BC patients. Using the TNMplot web-based tool (Bartha and Gyorffy 2021), Mann–Whitney analysis of TBC1D30 in normal or tumor tissue across 15,648 normal, 40,442 tumor, and 848 metastasis unique patient samples in 22 cancer types revealed that TBC1D30 expression was significantly higher in tumor samples compared to normal tissue in several cancer types including breast, while expression was lower in other cancer types (Fig. 4c). In sum, pan-cancer expression analysis demonstrated that TBC1D30 was a tumor associated candidate for future studies.

## Discussion

Understanding specific genetic modifiers and molecular mechanisms that drive BC tumor aggressiveness will improve our understanding and treatment of the disease. Genetic modifiers of BC development may interact directly with cancer-initiating genes (ie *p53* and *RB*), influence cancer cell intrinsic mechanisms of DNA repair or cell cycle regulation, or impact response to cancer cells by the tumor microenvironment such as angiogenesis or antitumor immunity to variably impact patient outcomes. The study presented was designed to determine the interaction of modifiers and causal genes in governing the heterogeneity of BC phenotypes in a preclinical model. Previous work by our group using the C3Tag TNBC model demonstrated that pregnancy, obesity, overweight, and weight loss impacted preneoplastic lesions (hyperplasia and DCIS) as well as cancer burden, progression, and response to therapy (Sundaram et al. 2013; Sundaram, Freemerman, et al. 2014; Sundaram, Le, et al. 2014; Cozzo et al. 2016; Qin et al. 2016). The C3Tag GEMM was enhanced herein by crossing it to the highly genetically diverse BXD family to aid in the discovery of genetic modifiers.

A novel model was created, the BXD-BC, which demonstrates heritable variation in multiple breast tumor phenotypes. Average tumor burden was reduced compared to the parent strain across all BXD-BC strains suggesting that the genetic variance introduced by intercrosses with various BXD strains affected both total tumor mass and multiplicity. Compared to novel BXD-BC F1, the parent C3Tag in the FVB strain displayed above-average latency and burden but lower survival and multiplicity which demonstrated that crosses into the BXD strains modulate susceptibility even in a GEMM. Thus, despite C3(1) Tag being a highly penetrant transgenically driven model, the cross to the BXD strains introduced variation, which has allowed for the identification of genetic variants. The intercross generated BXD-BC F1s with demonstrated *accelerated* tumor onset (i.e. mice with younger latency) or *delayed* tumor onset (i.e. older latency). Therefore, future studies using extremes of latency may aid in identifying the molecular underpinnings of pathways associated with BC traits. The most highly heritable traits were latency, endpoint, and multiplicity. When F1s from the 2 hybrids did not develop tumors, even when the mice were aged out until 1 year, survival became the greatest heritable trait. How the BXD-BC51 and BXD-BC79 F1 failed to present with mammary tumors is under investigation. Significant QTLs were identified in the BXD-BC F1 based on traits including tumor multiplicity and latency as well as histological qualities including the necrosis or the stroma and vascularity scores.

First, genetic modifiers of tumor multiplicity were examined, and the highest LOD was identified on Chr 16 after covariate analysis. Within the Chr 16 QTL interval, *cis*-eQTL analysis identified 4 candidate genes—*Slc15a2*, *Dtx3l*, *Rubcn*, and *Zdhhc23*—with expression in immune-related tissues and cells correlating positively or negatively with tumor multiplicity, suggesting complex regulatory mechanisms. PheWAS associations further support the relevance of SLC15A2, DTX3L, and ZDHHC23 in inflammation, DNA repair, and immune regulation, highlighting their potential roles in tumorigenesis. In sum, identification of 4 candidate genes associated with tumor multiplicity in the BXD-BC hybrids lends strength to this novel model given the links of the identified genes with previously identified variants in antitumor immunity, DNA repair, and human cancer-associated phenotypes.

In contrast to the many QTLs identified with tumor multiplicity, 1 significant QTL was identified on Chr 10 for tumor latency containing prioritized candidates *Gns*, *Tbc1d30*, and *Rassf3*. Functional evidence supports their roles: *GNS*, a lysosomal enzyme, influences cancer cell proliferation; *RASSF3*, a RAS effector, is frequently inactivated in tumors; and *TBC1D30*, a RAB-GTPase activator, interacts with ERα and is predicted to mediate tamoxifen- or fulvestrant-resistance in BC leading to poor outcomes (Vogel et al. 2015; Nassa et al. 2019; Selinger et al. 2022). Survival analyses showed that elevated *TBC1D30*, alone or in combination with *GNS* and *RASSF3*, correlated with reduced overall BC patient survival. Notably, *TBC1D30* is broadly overexpressed in tumors compared with normal tissue. Cross-species GWAS studies of mammals, including goats, sheep, and pigs, Matrangelo et *al*. and Bovo et *al*. demonstrated that all 3 genes of interest (*GNS*, *TBC1D30*, and *RASSF3*) were found in QTLs associated with fat deposition (or obesity) (Kudo et al. 2012; Donninger et al. 2016; Dhanaraman et al. 2020). Obesity is established to lead to 13 cancers and is strongly associated with worse outcomes and increased mortality (Lam et al. 2024). In sum, this novel BXD-BC hybrid model enabled the identification of 3 candidate genes associated with tumor latency which are also associated with prostate cancer and BC in humans and obesity across multiple species.

In summary, we evaluated 13 clinically relevant cancer phenotypes across the BXD-BC GEMMs, such as measures of tumor onset, multiplicity, burden, and survival. Among these, latency and multiplicity showed the highest heritability, with significant QTLs identified. Within these loci, we highlighted *Gns*, *Tbc1d30*, and *Rassf3* as poor prognostic markers supported by eQTL and PheWAS analyses. These phenotypes, together with their associated genetic drivers, provide a multidimensional framework for leveraging recombinant inbred GEMMs to dissect genetic modifiers of BC and explore mechanistic parallels with human BC. A limitation to be noted is that all of this work has been accomplished with a single GEMM. Using additional models such as the collaborative cross or diversity outbred families would complement this work (Chesler 2014; Abu Toamih Atamni et al. 2018). Another limitation is that we did not complete an extensive comparison of the parent GEMM to the various strains for histologic and genomic alterations. Indeed, future genomic work would include analyses on histologic traits, transcriptomic profiles, and global genomic alterations such as copy number changes in BXD-BC GEMMs that will elucidate therapeutic markers. Exploring additional developmental or pathway-related genes aside from cancer, as well as long noncoding RNAs and other non-protein-coding elements, may also hold promise for advancing our understanding and treatment strategies. Developmental genes or those affecting pathways tangentially linked to tumor biology (e.g. cell adhesion, immune regulation, and metabolism) may also play important roles. Our initial prioritization strategy emphasized known cancer genes to highlight clear translational relevance; however, we recognize the possibility to exponentially expand our focus (e.g. mitochondria, immunity, metabolism, developmental, and response to environment) to find interactions to cause phenotypes related to cancer. Lastly, in future studies, we aim to take advantage of this BXD GEMM to eventually examine both prostate and TNBC to look for commonalities such as androgen signaling. We and others have identified androgen receptor (AR), splice variants, and signaling in luminal androgen receptor (LAR) BC subtype which could be further dissected in the BXD-BC or prostate cancer model (Li et al. 2022; Asemota et al. 2023; Fernandez et al. 2023).

In conclusion, this report describes the novel BXD-BC model, wherein multiple QTLs and eQTLs were identified, which yielded candidate genes of interest associated with significantly heritable tumor traits. Candidate genes were demonstrated to be conserved in cancer or cancer-related phenotypes in PheWAS studies. Future work is necessary to further validate candidate genes as novel therapeutic targets to improve TNBC outcomes.

## Supplementary Material

jkaf238_Supplementary_Data

## Data Availability

The data generated in this study are available within the article and its supplementary data files or in GeneNetwork.org with indicated accession IDs: BXD_21526, BXD_21527, BXD_21528, BXD_21529, BXD_21530, BXD_21531, BXD_21532, BXD_21537, BXD_24398, BXD_24401, BXD_24402, BXD_24403, BXD_24404, BXD_24405, BXD_24406, BXD_24407, BXD_24408, BXD_24412, BXD_27513, BXD_27514, BXD_27515, BXD_27516, and BXD_27517. Supplemental material available at G3 online.
