# Supplementary material for: Identification of susceptibility loci using a novel murine model for triple-negative breast cancer
Source: G3 (Bethesda). 2025 Oct 10;16(2):jkaf238. doi: 10.1093/g3journal/jkaf238 (PMC12869084; doi:10.1093/g3journal/jkaf238)
Supplement: jkaf238_Supplementary_Data [file jkaf238_supplementary_data.zip › Supplemental_Figure_1_G3-2025-406194.pdf]

Supplemental Figure S1

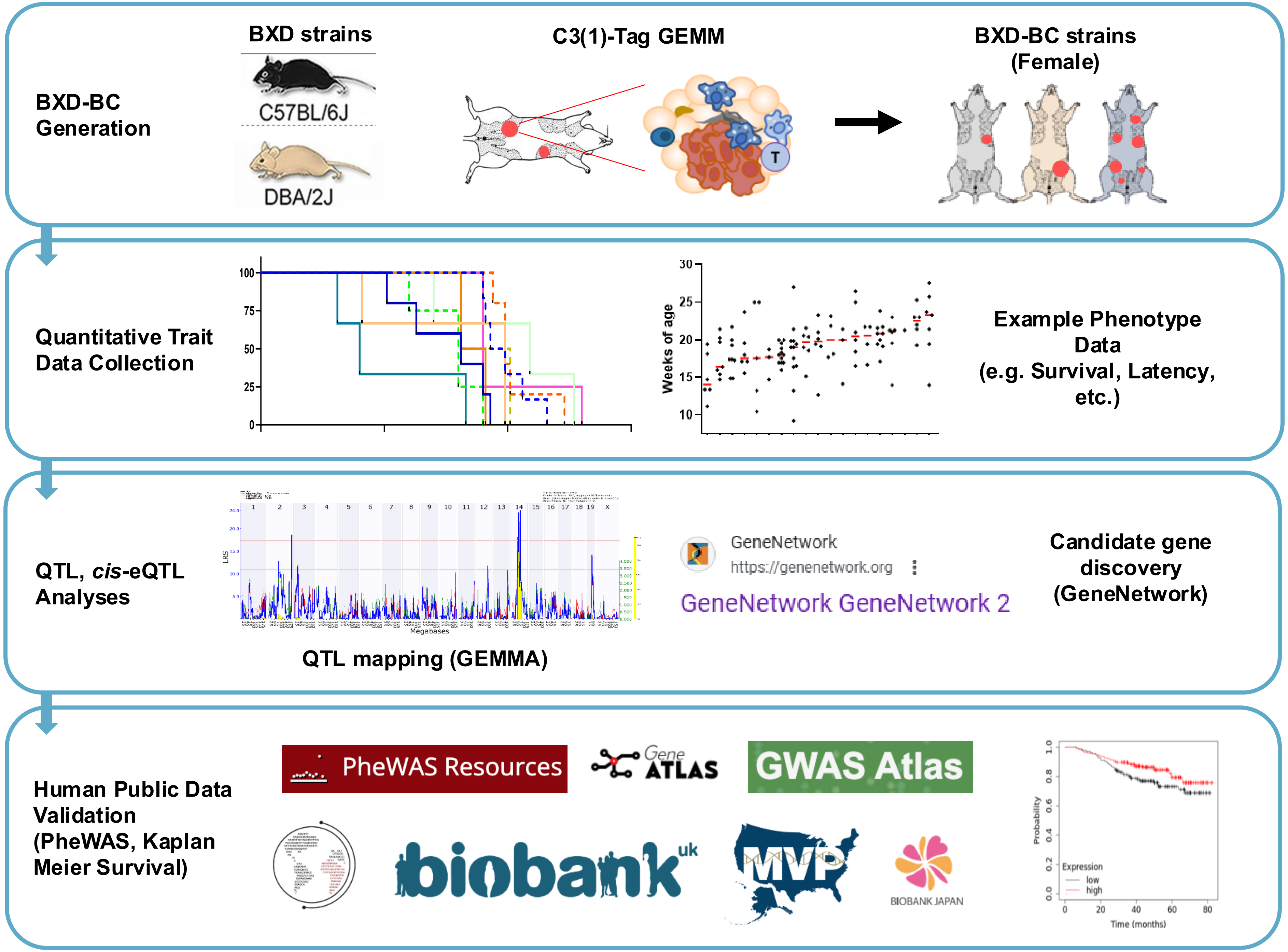

Supplemental Figure 1. Schematic of the study. Overview of the BXD-BC model study, including generation of BXD-BC strains, quantitative trait data collection, followed by quantitative trait loci (QTL), *cis*-eQTL analyses, and validation using human public datasets.
