# Supplementary material for: Identification of susceptibility loci using a novel murine model for triple-negative breast cancer
Source: G3 (Bethesda). 2025 Oct 10;16(2):jkaf238. doi: 10.1093/g3journal/jkaf238 (PMC12869084; doi:10.1093/g3journal/jkaf238)
Supplement: jkaf238_Supplementary_Data [file jkaf238_supplementary_data.zip › Supplemental_Figure_2_G3-2025-406194.pdf]

Supplemental Figure S2

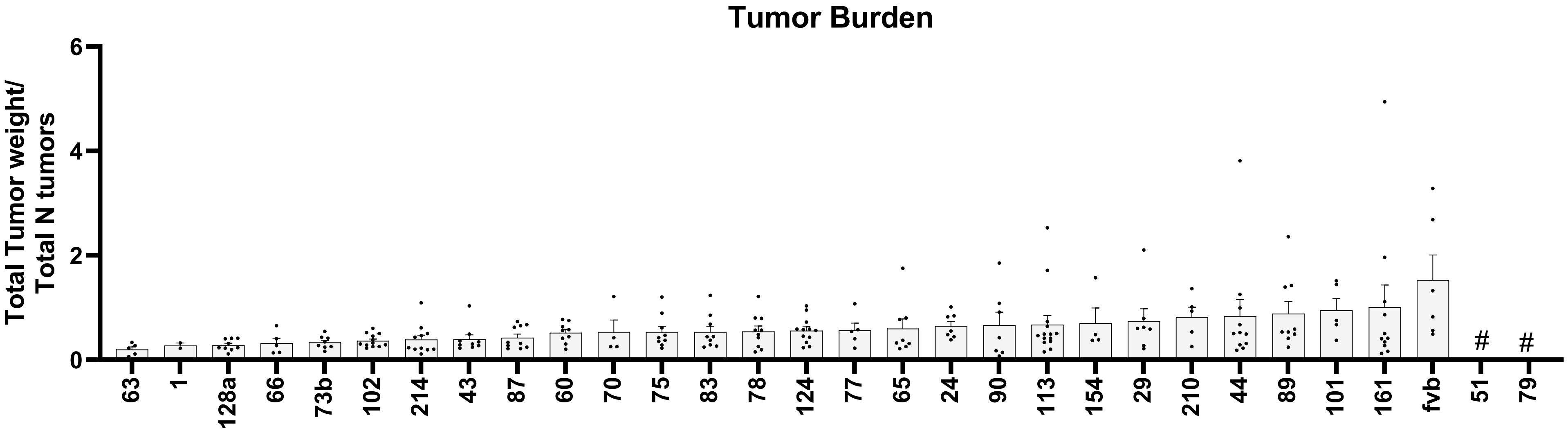

Supplemental Figure 2. Tumor burden demonstrates variability across F1 hybrids. Average tumor burden (total tumor weight/total N tumor (multiplicity)) is shown with BXD-BC strain on the X axis and each dot representing an individual mouse. The shaded bar represents the mean +/- SEM. "#" denotes F1s with no tumor development after 12 months.
