# Supplementary material for: Identification of susceptibility loci using a novel murine model for triple-negative breast cancer
Source: G3 (Bethesda). 2025 Oct 10;16(2):jkaf238. doi: 10.1093/g3journal/jkaf238 (PMC12869084; doi:10.1093/g3journal/jkaf238)
Supplement: jkaf238_Supplementary_Data [file jkaf238_supplementary_data.zip › Supplemental_Figure_3_G3-2025-406194.pdf]

Supplemental Figure S3

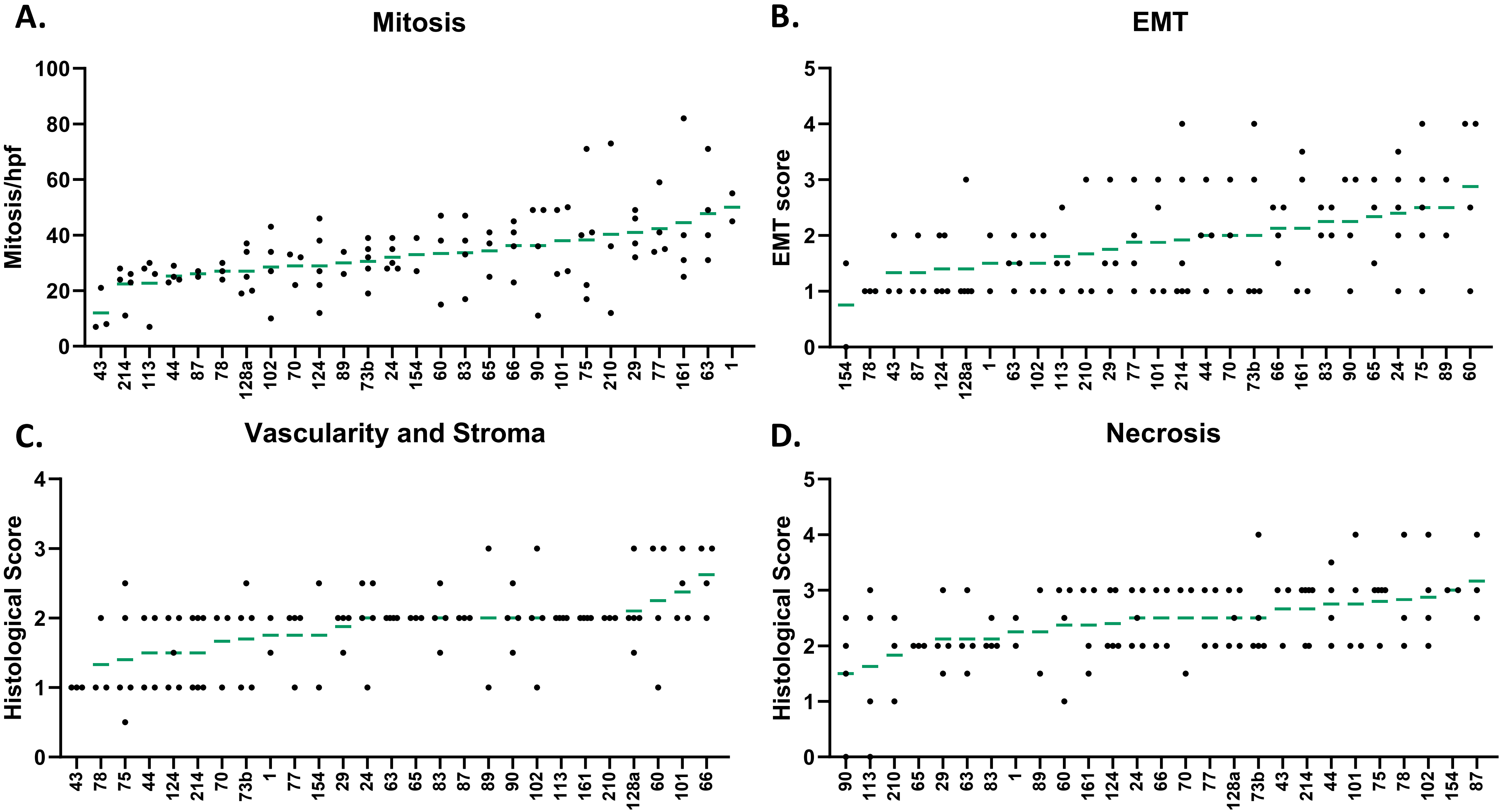

Supplemental Figure 3. Tumor histologic analysis revealed variability phenotypes across F1 hybrids. Tumors were stained by H and E and analyzed by a veterinary pathologist, Dr. Robert Read, DVM. Analysis for each trait was averaged from over three randomly selected regions of interest in tumor H and E sections and a score was generated. Data were ranked by mean (green line) with each dot representing one mouse tumor. **A**. Mitosis per high-powered field (hpf) was quantified in 40X images. **B**. Epithelial to mesenchymal transition (EMT) type histology was scored from 0 to 4: 0=no pleomorphism; 1=subepithelial pleomorphism; 2=faint streaming; 3=distinct frequent streaming; and 4=distinct sarcomatous transition. **C**. Vascularity and stromal content were scored from 0-5: 0=none; 1=minimal stroma; 2=mild; 3=moderate; 4=heavy; and 5=scirrhous. **D**. Necrosis of the tumor was scored from none to cavitary tumor loss across a five-point scale.
