# Supplementary material for: Identification of susceptibility loci using a novel murine model for triple-negative breast cancer
Source: G3 (Bethesda). 2025 Oct 10;16(2):jkaf238. doi: 10.1093/g3journal/jkaf238 (PMC12869084; doi:10.1093/g3journal/jkaf238)
Supplement: jkaf238_Supplementary_Data [file jkaf238_supplementary_data.zip › Supplemental_Figure_4_G3-2025-406194.pdf]

Supplemental Figure S4

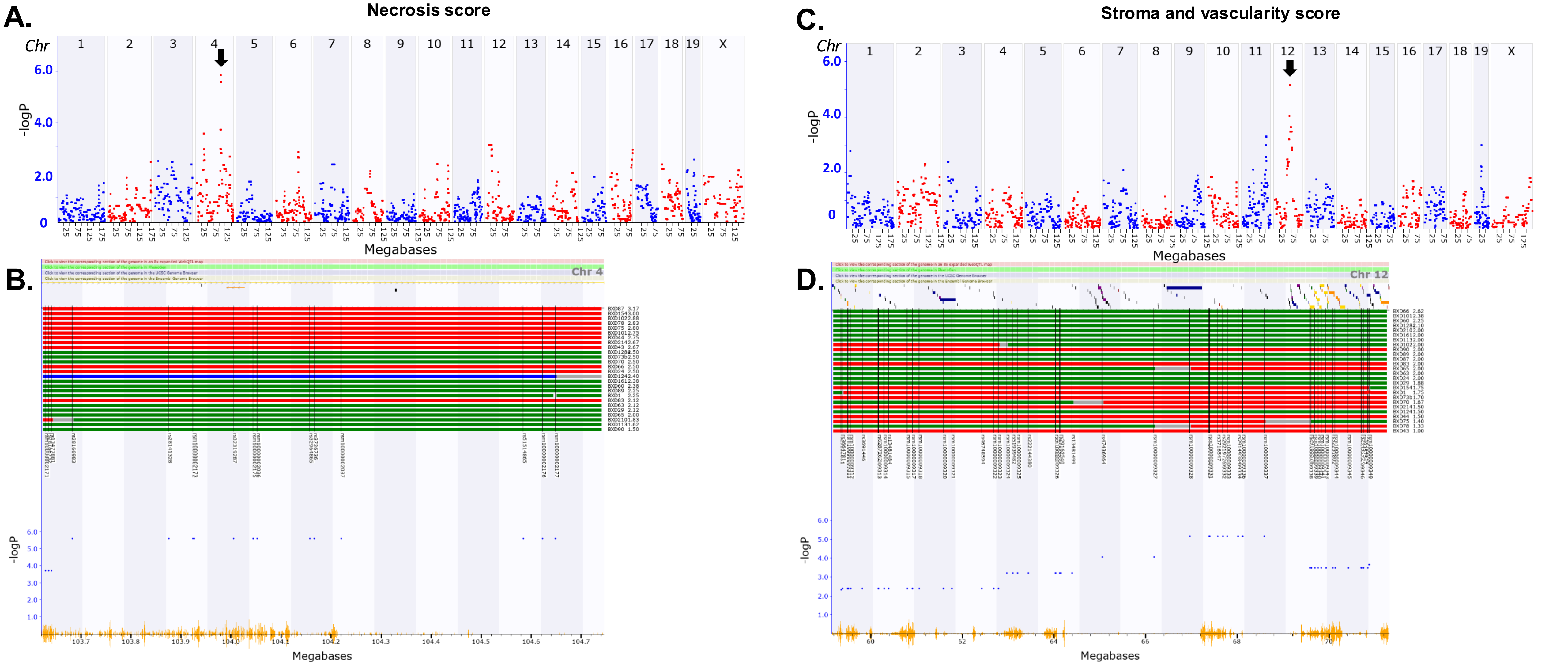

Supplemental Figure 4. A significant quantitative trait locus (QTL) for tumor histological scores was identified on Chromosomes 4 and 12. QTLs associated with necrosis as scored by histology were examined in GEMMA. **A.** Genome wide QTL plot demonstrates the logarithm of the odds (LOD score (blue line) at each marker across the genome with chromosomes noted on the X- axis for tumor necrosis. The genome-wide significant threshold is indicated by the pink line (-logP = 3.82). The genome-wide suggestive threshold of -logP = 2.94 is indicated by the grey line. **B**. Zoomed image of the 1.5 LOD-drop confidence interval for the QTL on chromosome 4 is shown with megabases on the X-axis. Red and green horizontal bars at the top of the figure represent the haplotypes at the position – green is the D-like DBA/2J-derived haplotype and red is the B-like C57BL/6J-derived haplotype. BXD-BC hybrids are aligned on the top left with # indicating necrosis score. On the Y-axis, blue dots represent the -log(p) linkage between a marker and tumor latency. Segregating SNPs in the BXD family is shown by the orange Seismograph at the bottom. **C.** Genome-wide QTL plot for histological score for stroma and vascularity, with genome-wide significant threshold is indicated by pink line (-logP = 3.69). The genome-wide suggestive threshold of -logP = 2.78 is indicated by the grey line. **D**. Zoomed image of the 1.5 LOD-drop confidence interval for the QTL on Chr 12 is shown with megabases on the X-axis. BXD-BC F1s are aligned on top left with # indicating histologic score for stroma and vascularity.
