# Supplementary material for: Identification of susceptibility loci using a novel murine model for triple-negative breast cancer
Source: G3 (Bethesda). 2025 Oct 10;16(2):jkaf238. doi: 10.1093/g3journal/jkaf238 (PMC12869084; doi:10.1093/g3journal/jkaf238)
Supplement: jkaf238_Supplementary_Data [file jkaf238_supplementary_data.zip › Supplemental_Figure_5_G3-2025-406194.pdf]

Supplemental Figure S5

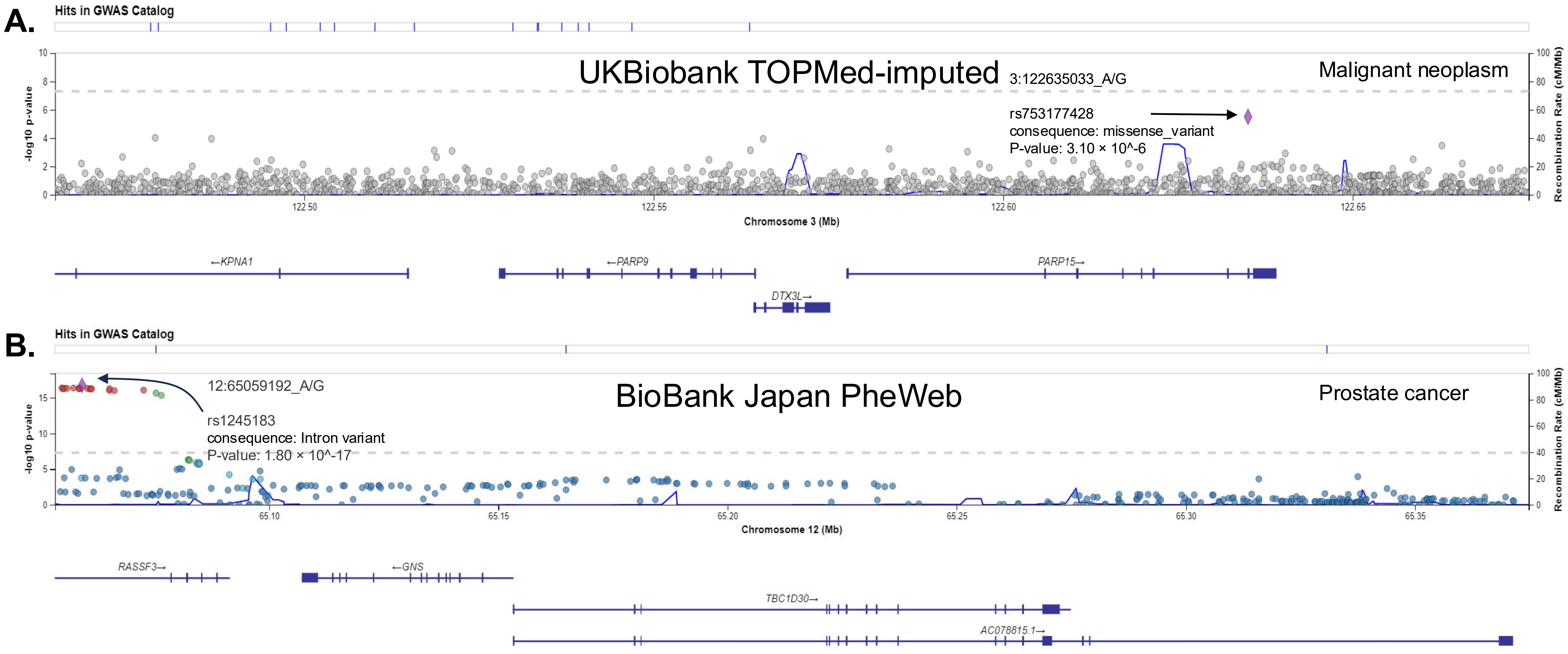

Supplemental Figure 5. PheWAS study identified candidate gene variants mediating tumor multiplicity and latency. A. In the University of Michigan PheWAS study, *DTX3L* was associated with 'Malignant neoplasm, other' with p value= 3.10e-06, influenced by *PARP15* missense variant. B. BioBank Japan reports *RASSF3* intro variants (rs7968403 & rs1245183) to influence nearby *TBC1D30* and *GNS* expressions associated with 'Prostate cancer' p value=1.80e-17.
