# Supplementary material for: Identification of susceptibility loci using a novel murine model for triple-negative breast cancer
Source: G3 (Bethesda). 2025 Oct 10;16(2):jkaf238. doi: 10.1093/g3journal/jkaf238 (PMC12869084; doi:10.1093/g3journal/jkaf238)
Supplement: jkaf238_Supplementary_Data [file jkaf238_supplementary_data.zip › Supplemental_Material_Legends_G3-2025-406194.docx]

**SUPPLEMENTAL FIGURE LEGENDS**

**Supplemental Figure 1. Schematic of the study.** Overview of the BXD-BC model study, including generation of BXD-BC strains, quantitative trait data collection, followed by quantitative trait loci (QTL), *cis*-eQTL analyses, and validation using human public datasets.

**Supplemental Figure 2.** **Tumor burden demonstrates variability across F1 hybrids.** Average tumor burden (total tumor weight/total N of tumor (multiplicity)) is shown with BXD-BC strain on the X axis and each dot representing an individual mouse. The shaded bar represents the mean +/- SEM. “#” denotes F1s with no tumor development after 12 months.

**Supplemental Figure 3. Tumor histologic analysis revealed variability phenotypes across F1 hybrids.** Tumors were stained by H and E and analyzed by a veterinary pathologist, Dr. Robert Read, DVM. Analysis for each trait was averaged from over three randomly selected regions of interest in tumor H and E sections and a score was generated. Data were ranked by mean (green line) with each dot representing one mouse tumor. **A**. Mitosis per high-powered field (hpf) was quantified in 40X images. **B**. Epithelial to mesenchymal transition (EMT) type histology was scored from 0 to 4: 0=no pleomorphism; 1=subepithelial pleomorphism; 2=faint streaming; 3=distinct frequent streaming; and 4=distinct sarcomatous transition. **C**. Vascularity and stromal content were scored from 0-5: 0=none; 1=minimal stroma; 2=mild; 3=moderate; 4=heavy; and 5=scirrhous. **D**. Necrosis of the tumor was scored from none to cavitary tumor loss across a five-point scale.

**Supplemental Figure 4.** **A significant quantitative trait locus (QTL) for tumor histological scores was identified on Chromosomes 4 and 12**. QTLs associated with necrosis as scored by histology were examined in GEMMA. **A.** Genome wide QTL plot demonstrates the logarithm of the odds (LOD score (blue line) at each marker across the genome with chromosomes noted on the X- axis for tumor necrosis. The genome-wide significant threshold is indicated by the pink line (-logP = 3.82). The genome-wide suggestive threshold of -logP = 2.94 is indicated by the grey line. **B**. Zoomed image of the 1.5 LOD-drop confidence interval for the QTL on chromosome 4 is shown with megabases on the X-axis. Red and green horizontal bars at the top of the figure represent the haplotypes at the position – green is the D-like DBA/2J-derived haplotype and red is the B-like C57BL/6J-derived haplotype. BXD-BC hybrids are aligned on the top left with # indicating necrosis score. On the Y-axis, blue dots represent the -log(p) linkage between a marker and tumor latency. Segregating SNPs in the BXD family is shown by the orange Seismograph at the bottom. **C.** Genome-wide QTL plot for histological score for stroma and vascularity, with genome-wide significant threshold is indicated by pink line (-logP = 3.69). The genome-wide suggestive threshold of -logP = 2.78 is indicated by the grey line. **D**. Zoomed image of the 1.5 LOD-drop confidence interval for the QTL on Chr 12 is shown with megabases on the X-axis. BXD-BC F1s are aligned on top left with # indicating histologic score for stroma and vascularity.

**Supplemental Figure 5. PheWAS study identified candidate gene variants mediating tumor multiplicity and latency. A.** In the University of Michigan PheWAS study, *DTX3L* was associated with ‘Malignant neoplasm, other’ with p value= 3.10e-06, influenced by *PARP15* missense variant. B. BioBank Japan reports *RASSF3* intro variants (rs7968403 & rs1245183) to influence nearby *TBC1D30* and *GNS* expressions associated with ‘Prostate cancer’ p value=1.80e-17.

**SUPPLEMENTAL TABLES**

**Supplemental Table 1.** **BXD-BC trait IDs in GeneNetwork.** BXD-BC record ID in GeneNetwork listed for each trait based on tumor characteristics and histologic analysis.

**Supplemental Table 2**. **Search engines used for Human Phenome-Wide Association Analysis.**

**Supplemental Table 3. BXD Haplotype in 28 Strains.** Genotype data downloaded GeneNetwork ([Ashbrook *et al.* 2022](#_ENREF_7)). For each marker, alleles were coded as B, D, or H (heterozygous).

**Supplemental Table 4**. **BXD-BC Hybrids Generated. ID and mice per cross are indicated.**

**Supplemental Table 5. Multiple Tumor Traits Display Significant Heritability in BXD-BC Hybrids**. Heritability (*h^2^*) and significance of strain effect (p) are shown tumor traits collected for N=28 BXD-BC crosses, with an average of 8 replicates per hybrid. Strain effect was tested by ANOVA. Hybrids that did not develop tumors (BXD-BC51 and BXD-BC79) were included. Bold indicates significance P<0.05.

**Supplemental Table 6. Chromosome 16 QTL Protein Coding Genes associated with Tumor Multiplicity.** An interval generated in GEMMA for chr16 (26.4-44.1 Mb) contains 80 annotated genes and open reading frames (ORFs), of which 40 were protein coding with gene symbol, description, start point, length, and variant or single nucleotide polymorphism (SNP) counts and density reported. Of the protein-coding candidate genes, 31 gene variants were predicted to alter protein function or splice regions in 11 genes (bolded).

**Supplemental Table 7. Chromosome 10 QTL Protein Coding Genes.** An interval generated in GEMMA for chr10 (117.3- 121.5 Mb) contains 121 annotated genes and open reading frames (ORFs), of which 27 were protein coding with gene symbol, description, start point, length, and variant or single nucleotide polymorphism (SNP) counts and density reported. Bolded genes are of particular interest.

**Supplemental Table 8. Tumor Histology Traits Display Insignificant Heritability in all BXD-BC Hybrids.** Heritability (*h^2^*) and significance of strain effect (p) are shown tumor traits collected for N=26 BXD-BC crosses, with an average of 8 replicates per hybrid. Strain effect was tested by ANOVA. Hybrids that did not develop tumors were not included. Scored histology was averaged from over 3 randomly selected regions of interest in tumor H+E section.

**Supplemental Table 9. Chromosome 4 QTL Protein Coding Genes for Necrosis**. An interval generated in GEMMA for chr4 (103.64-105.24 Mb) contains 23 annotated genes and open reading frames (ORFs), of which 6 were protein coding with gene symbol, description, start point, length, and variant or single nucleotide polymorphisms (SNP) counts and density reported.

**Supplemental Table 10. Chromosome 12 QTL Protein Coding Genes for Stroma and Vascularity.** An interval generated in GEMMA for chr12 64.387817- 69.575599 Mb) contains 172 annotated genes and open reading frames (ORFs), of which 43 were protein coding with gene symbol, description, start point, length, and variant or single nucleotide polymorphisms (SNP) counts and density reported.
