# Supplementary material for: Identification of susceptibility loci using a novel murine model for triple-negative breast cancer
Source: G3 (Bethesda). 2025 Oct 10;16(2):jkaf238. doi: 10.1093/g3journal/jkaf238 (PMC12869084; doi:10.1093/g3journal/jkaf238)
Supplement: jkaf238_Supplementary_Data [file jkaf238_supplementary_data.zip › Supplemental_Table_1_G3-2025-406194.pdf]

Supplemental Table 1. BXD-BC record ID in GeneNetwork listed for each trait based on tumor characteristics and histologic analysis.

BXD Record ID#	Phenotype, Description, and Units
27516	Cancer: Breast cancer inflammation, tumor elicited inflammation, 0 = none, 1= minimal, 2= mild, 3 moderate, 4 heavy, 5=extensive inflammation, in female F1 (female BXD crossed to male C3 (1)-TAg (JAX Stock 013591: FVB-Tg(C3-1TAg)cJeg/JegJ, SV40 T/t-antig
21537	Cancer: Breast cancer age at morbidity (euthanasia) in female F1 (female BXD crossed to male C3 (1)-TAg (JAX Stock 013591: FVB-Tg(C3-1TAg)cJeg/JegJ, SV40 T/t-antigen) strain mean including never developed [age in weeks]
24401	Cancer: Breast cancer age at morbidity (euthanasia) in female F1 (female BXD crossed to male C3 (1)-TAg (JAX Stock 013591: FVB-Tg(C3-1TAg)cJeg/JegJ, SV40 T/t-antigen) strain mean including strains that never developed tumors [age in days]
21527	Cancer: Breast cancer age at morbidity (euthanasia) in female F1 (female BXD crossed to male C3 (1)-TAg (JAX Stock 013591: FVB-Tg(C3-1TAg)cJeg/JegJ, SV40 T/t-antigen) strain mean [age in days]
27513	Cancer: Breast cancer Epithelial-mesenchymal transition (EMT) on 5-point scale, 0 = no pleomorphism, 1 = subepithelial pleomorphism, 2 = faint streaming, 3 = distinct frequent streaming, 4 = distinct sarcomatous transition seen, in female F1 (female BXD c
21529	Cancer: Breast cancer Mean volume of first tumour in female F1 (female BXD crossed to male C3 (1)-TAg (JAX Stock 013591: FVB-Tg(C3-1TAg)cJeg/JegJ, SV40 T/t-antigen) strain mean (mm^3).
27517	Cancer: Breast cancer mitosis, as number of mitoses per high-power field (40X) averaged across 3 or more areas of tumor in female F1 (female BXD crossed to male C3 (1)-TAg (JAX Stock 013591: FVB-Tg(C3-1TAg)cJeg/JegJ, SV40 T/t-antigen) strain mean
27515	Cancer: Breast cancer necrosis, prevalence of necrosis and cavitary tumor loss, 0 = none, 1= minimal, 2= mild, 3 moderate, 4 heavy, 5=extensive necrosis, in female F1 (female BXD crossed to male C3 (1)-TAg (JAX Stock 013591: FVB-Tg(C3-1TAg)cJeg/JegJ, SV40
21528	Cancer: Breast cancer number of tumors at morbidity (euthanasia) in female F1 (female BXD crossed to male C3 (1)-TAg (JAX Stock 013591: FVB-Tg(C3-1TAg)cJeg/JegJ, SV40 T/t-antigen) Strain mean (n)
24403	Cancer: Breast cancer number of tumors at morbidity (euthanasia) in female F1 (female BXD crossed to male C3 (1)-TAg (JAX Stock 013591: FVB-Tg(C3-1TAg)cJeg/JegJ, SV40 T/t-antigen) strain mean including strains that never developed tumors (n)
21530	Cancer: Breast cancer total tumor volume at morbidity (euthanasia) in female F1 (female BXD crossed to male C3 (1)-TAg (JAX Stock 013591: FVB-Tg(C3-1TAg)cJeg/JegJ, SV40 T/t-antigen) -Strain mean (mm^3)
24405	Cancer: Breast cancer total tumor volume at morbidity (euthanasia) in female F1 (female BXD crossed to male C3 (1)-TAg (JAX Stock 013591: FVB-Tg(C3-1TAg)cJeg/JegJ, SV40 T/t-antigen) strain mean including strains that never developed tumors with strains th
21532	Cancer: Breast cancer total weight of tumours at morbidity (euthanasia) in female F1 (female BXD crossed to male C3 (1)-TAg (JAX Stock 013591: FVB-Tg(C3-1TAg)cJeg/JegJ, SV40 T/t-antigen) - strain mean (mg)
24407	Cancer: Breast cancer total weight of tumours at morbidity (euthanasia) in female F1 (female BXD crossed to male C3 (1)-TAg (JAX Stock 013591: FVB-Tg(C3-1TAg)cJeg/JegJ, SV40 T/t-antigen) strain mean including strains that never developed tumors as 0 (mg)
24402	Cancer: Breast cancer tumor development latency in female F1 (female BXD crossed to male C3 (1)-TAg (JAX Stock 013591: FVB-Tg(C3-1TAg)cJeg/JegJ, SV40 T/t-antigen) strain mean including strains that never developed tumors as 52 weeks [age in weeks]
21526	Cancer: Breast cancer tumor development latency in female F1 (female BXD crossed to male C3 (1)-TAg (JAX Stock 013591: FVB-Tg(C3-1TAg)cJeg/JegJ, SV40 T/t-antigen) strain mean [age in weeks]
24412	Cancer: Breast cancer tumor time between first detection and euthanasia in female F1 (female BXD crossed to male C3 (1)-TAg (JAX Stock 013591: FVB-Tg(C3-1TAg)cJeg/JegJ, SV40 T/t-antigen) strain mean including strains that never developed tumors [days]
24408	Cancer: Breast cancer tumor, one tumor greater than 2cm at death in female F1 (female BXD crossed to male C3 (1)-TAg (JAX Stock 013591: FVB-Tg(C3-1TAg)cJeg/JegJ, SV40 T/t-antigen) strain mean including strains that never developed tumors as 0 [n]
24398	Cancer: Breast cancer tumor, one tumor greater than 2cm at morbidity (euthanasia) in female F1 (female BXD crossed to male C3 (1)-TAg (JAX Stock 013591: FVB-Tg(C3-1TAg)cJeg/JegJ, SV40 T/t-antigen). Strain mean [n]
27514	Cancer: Breast cancer vascularity and stroma score, 0 = none, 1 = minimal stroma, 2= mild, 3 = moderate, 4 = heavy, 5 = scirrhous, in female F1 (female BXD crossed to male C3 (1)-TAg (JAX Stock 013591: FVB-Tg(C3-1TAg)cJeg/JegJ, SV40 T/t-antigen) strain me
24404	Cancer: Breast cancer volume of first tumor in female F1 (female BXD crossed to male C3 (1)-TAg (JAX Stock 013591: FVB-Tg(C3-1TAg)cJeg/JegJ, SV40 T/t-antigen) strain strain mean including strains that never developed tumors with strains that never develop
21531	Cancer: Breast cancer weight of first tumour at morbidity (euthanasia) in female F1 (female BXD crossed to male C3 (1)-TAg (JAX Stock 013591: FVB-Tg(C3-1TAg)cJeg/JegJ, SV40 T/t-antigen) - strain mean (mg)
24406	Cancer: Breast cancer weight of first tumour at morbidity (euthanasia) in female F1 (female BXD crossed to male C3 (1)-TAg (JAX Stock 013591: FVB-Tg(C3-1TAg)cJeg/JegJ, SV40 T/t-antigen) strain mean including strains that never developed tumors as 0 (mg)
