# Supplementary material for: Identification of susceptibility loci using a novel murine model for triple-negative breast cancer
Source: G3 (Bethesda). 2025 Oct 10;16(2):jkaf238. doi: 10.1093/g3journal/jkaf238 (PMC12869084; doi:10.1093/g3journal/jkaf238)
Supplement: jkaf238_Supplementary_Data [file jkaf238_supplementary_data.zip › Supplemental_Table_10_G3-2025-406194.pdf]

Supplemental Table 10. Chromosome 12 QTL Protein Coding Genes for Stroma and Vascularity. An interval generated in GEMMA for chr12 64.387817- 69.575599 Mb) contains 172 annotated genes and open reading frames (ORFs), of which 43 were protein coding with gene symbol, description, start point, length, and variant or single nucleotide polymorphisms (SNP) counts and density reported.

Symbol	Gene Description	Mb Start	Length (Kb)	SNP Count	SNP Density
Mia2	MIA SH3 domain ER export factor 2	59.095582	94.64	22	0.23
Fbxo33	F-box protein 33	59.200655	21.31	11	0.52
Lrfn5	leucine rich repeat and fibronectin type III domain containing 5	61.522851	335.08	70	0.21
Spanxn4	SPANX family, member N4	62.687787	0.55	0	0.00
Fscb	fibrous sheath CABYR binding protein	64.471333	3.57	0	0.00
Klhl28	kelch-like 28	64.941486	24.10	0	0.00
Togaram1	TOG array regulator of axonemal microtubules 1	64.965576	57.00	3	0.05
Fkbp3	FK506 binding protein 3	65.062432	11.51	0	0.00
Fancm	Fanconi anemia, complementation group M	65.074110	57.73	2	0.03
Mis18bp1	MIS18 binding protein 1	65.132734	39.85	0	0.00
Wdr20rt	WD repeat domain 20, retrogene	65.225517	2.94	0	0.00
Rpl10l	ribosomal protein L10-like	66.283379	1.02	0	0.00
Mdga2	MAM domain containing glycosylphosphatidylinositol anchor 2	66.459419	763.13	91	0.12
Rps29	ribosomal protein S29	69.157722	1.46	0	0.00
Lrr1	leucine rich repeat protein 1	69.168814	10.20	7	0.69
Rpl36al	ribosomal protein L36A-like	69.182734	1.33	0	0.00
Mgat2	mannoside acetylglucosaminyltransferase 2	69.184158	2.62	0	0.00
Dnaaf2	dynein, axonemal assembly factor 2	69.189087	11.00	1	0.09
Pole2	polymerase (DNA directed), epsilon 2 (p59 subunit)	69.201773	26.43	8	0.30
Klhdc1	kelch domain containing 1	69.227729	56.92	17	0.30
Klhdc2	kelch domain containing 2	69.296681	14.01	4	0.29
Nemf	nuclear export mediator factor	69.311541	45.66	1	0.02
Arf6	ADP-ribosylation factor 6	69.372150	3.83	0	0.00
Vcpkmt	valosin containing protein lysine (K) methyltransferase	69.576735	6.28	12	1.91
Sos2	SOS Ras/Rho guanine nucleotide exchange factor 2	69.583761	98.09	219	2.23
L2hgdh	L-2-hydroxyglutarate dehydrogenase	69.690436	34.44	76	2.21
Dmac2l	distal membrane arm assembly complex 2 like	69.724913	19.75	24	1.22
Cdkl1	cyclin-dependent kinase-like 1 (CDC2-related kinase)	69.745167	46.03	63	1.37
Map4k5	mitogen-activated protein kinase kinase kinase kinase 5	69.803757	89.41	238	2.66
Atl1	atlastin GTPase 1	69.893105	70.98	181	2.55
Sav1	salvador family WW domain containing 1	69.965009	23.13	56	2.42
Nin	ninein	70.011435	102.38	403	3.94
Abhd12b	abhydrolase domain containing 12B	70.154124	35.97	128	3.56
Pygl	liver glycogen phosphorylase	70.190815	36.87	141	3.82
Trim9	tripartite motif-containing 9	70.244533	103.34	104	1.01
Tmx1	thioredoxin-related transmembrane protein 1	70.453154	14.47	3	0.21
Frmd6	FERM domain containing 6	70.825456	76.78	10	0.13
Actr10	ARP10 actin-related protein 10	70.937857	26.86	0	0.00
Psma3	proteasome subunit alpha 3	70.969283	26.59	2	0.08
Arid4a	AT rich interactive domain 4A (RBP1-like)	71.015081	84.27	11	0.13
Tomm20l	translocase of outer mitochondrial membrane 20-like	71.111428	11.79	5	0.42
Timm9	translocase of inner mitochondrial membrane 9	71.123172	13.54	26	1.92
Dact1	dishevelled-binding antagonist of beta-catenin 1	71.309884	10.22	48	4.70
