# Supplementary material for: Identification of susceptibility loci using a novel murine model for triple-negative breast cancer
Source: G3 (Bethesda). 2025 Oct 10;16(2):jkaf238. doi: 10.1093/g3journal/jkaf238 (PMC12869084; doi:10.1093/g3journal/jkaf238)
Supplement: jkaf238_Supplementary_Data [file jkaf238_supplementary_data.zip › Supplemental_Table_2_G3-2025-406194.pdf]

Supplementary Table 2. List of search engines used for Human Phenome-Wide Association Analysis		
**Search Engines**	**Website Links**	**Citations**
Global Biobank Engine	https://biobankengine.stanford.edu/	Global Biobank Engine, Stanford, CA (URL: http://gbe.stanford.edu).
Gene Atlas	http://geneatlas.roslin.ed.ac.uk/	Oriol Canela-Xandri, Konrad Rawlik and Albert Tenesa. An atlas of genetic associations in UK Biobank. Nature Genetics (2018). 50, 1593–1599.
PheWAS Catalog	https://phewascatalog.org/	Denny JC, Bastarache L, Ritchie MD et al. Systematic comparison of phenome-wide association study of electronic medical record data and genome-wide association study data. Nat Biotechnol. 2013 Dec;31(12):1102-10.
UKBiobank TOPMed-imputed	http://pheweb.sph.umich.edu/	Gagliano Taliun, S.A., VandeHaar, P. et al. Exploring and visualizing large-scale genetic associations by using PheWeb. Nat Genet 52, 550–552 (2020).
GWAS Atlas	https://atlas.ctglab.nl/PheWAS	Watanabe, K. et al. A global overview of pleiotropy and genetic architecture in complex traits. Nat. Genet. 51, 1339-1348 (2019). PMID: 31427789.
BioBank Japan PheWeb	https://pheweb.jp/	Sakaue, S., Kanai, M., Tanigawa, Y. et al. A cross-population atlas of genetic associations for 220 human phenotypes. Nat Genet 53, 1415–1424 (2021).
