# Supplementary material for: Identification of susceptibility loci using a novel murine model for triple-negative breast cancer
Source: G3 (Bethesda). 2025 Oct 10;16(2):jkaf238. doi: 10.1093/g3journal/jkaf238 (PMC12869084; doi:10.1093/g3journal/jkaf238)
Supplement: jkaf238_Supplementary_Data [file jkaf238_supplementary_data.zip › Supplemental_Table_4_G3-2025-406194.pdf]

Supplemental Table 4. BXD-BC Hybrids Generated. ID and mice per cross are indicated.

BXD-BC ID	# mice per hybrid
1	2
24	7
29	7
43	9
44	11
51	7
60	9
63	5
65	8
66	5
70	4
75	9
78	10
79	7
83	9
87	10
89	9
90	7
101	5
102	12
113	14
124	12
154	4
161	11
210	5
214	11
128a	9
73b	8
FVB	6
