# Supplementary material for: Identification of susceptibility loci using a novel murine model for triple-negative breast cancer
Source: G3 (Bethesda). 2025 Oct 10;16(2):jkaf238. doi: 10.1093/g3journal/jkaf238 (PMC12869084; doi:10.1093/g3journal/jkaf238)
Supplement: jkaf238_Supplementary_Data [file jkaf238_supplementary_data.zip › Supplemental_Table_5_G3-2025-406194.pdf]

Supplemental Table 5. Multiple Tumor Traits Display Significant Heritability in BXD-BC Hybrids. Heritability (h2) and significance of strain effect (p) are shown tumor traits collected for N=28 BXD-BC crosses, with an average of 8 replicates per hybrid. Strain effect was tested by ANOVA. Hybrids that did not develop tumors (BXD-BC51 and BXD-BC79) were included. Bold indicates significance P<0.05.

Phenotype (Trait)	h^2	p-value
Survival (Time from latency to endpoint)	0.970	**1.48E-134**
Age at endpoint	0.926	**8.90E-97**
Tumor latency	0.524	**9.63E-19**
Multiplicity	0.483	**1.38E-16**
Total tumor weight	0.317	**6.42E-07**
Total tumor volume	0.303	**2.74E-06**
Endpoint 1 tumor >2cm	0.203	**0.011**
Endpoint 3 tumors >1cm	0.197	**0.017**
T1 tumor weight	0.175	0.060
T1 volume	0.170	0.078
T1 tumor location	0.138	0.359
