# Supplementary material for: Identification of susceptibility loci using a novel murine model for triple-negative breast cancer
Source: G3 (Bethesda). 2025 Oct 10;16(2):jkaf238. doi: 10.1093/g3journal/jkaf238 (PMC12869084; doi:10.1093/g3journal/jkaf238)
Supplement: jkaf238_Supplementary_Data [file jkaf238_supplementary_data.zip › Supplemental_Table_6_G3-2025-406194.pdf]

Supplementary Table 6. Chromosome 16 QTL Protein Coding Genes associated with Tumor Multiplicity. An interval generated in GEMMA for chr16 (26.4-44.1 Mb) contains 80 annotated genes and open reading frames (ORFs), of which 40 were protein coding with gene symbol, description, start point, length, and variant or single nucleotide polymorphism (SNP) counts and density reported. Of the protein-coding candidate genes, 31 gene variants were predicted to alter protein function or splice regions in 11 genes (bolded).

Symbol	Gene Description	Mb Start	Length (Kb)	SNP Count	SNP Density
Heg1	heart development protein with EGF-like domains 1	33.684384	87.19	11	0.13
Muc13	mucin 13, epithelial transmembrane	33.794037	25.89	1	0.04
Itgb5	integrin beta 5	33.829665	119.67	152	1.27
Umps	uridine monophosphate synthetase	33.954782	12.26	4	0.33
Kalrn	kalirin, RhoGEF kinase	33.969073	604.20	1186	1.96
Ropn1	ropporin, rhophilin associated protein 1	34.649832	28.78	70	2.43
Ccdc14	**coiled-coil domain containing 14**	34.690557	34.65	11	0.32
Mylk	**myosin, light polypeptide kinase**	34.745199	257.24	402	1.56
Hacd2	3-hydroxyacyl-CoA dehydratase 2	35.022421	86.77	103	1.19
Adcy5	adenylate cyclase 5	35.154494	151.25	19	0.13
Sec22a	SEC22 homolog A, vesicle trafficking protein	35.311131	52.80	7	0.13
Pdia5	protein disulfide isomerase associated 5	35.397308	93.61	110	1.18
Sema5b	**semaphorin 5B**	35.541154	123.11	211	1.71
Dirc2	**solute carrier family 49 member 4**	35.694062	75.31	175	2.32
Hspbap1	**Hspb associated protein 1**	35.769726	58.74	10	0.17
Parp14	**poly (ADP-ribose) polymerase family, member 14**	35.831892	39.63	56	1.41
Dtx3l	**deltex 3-like, E3 ubiquitin ligase**	35.926515	12.51	55	4.40
Parp9	**poly (ADP-ribose) polymerase family, member 9**	35.93847	34.15	202	5.91
Kpna1	karyopherin (importin) alpha 1	35.983288	53.85	14	0.26
Wdr5b	**WD repeat domain 5B**	36.04119	1.78	0	0.00
Fam162a	family with sequence similarity 162, member A	36.043844	27.72	3	0.11
Ccdc58	coiled-coil domain containing 58	36.07166	20.46	13	0.64
Csta1	cystatin A1	36.119946	11.24	7	0.62
Stfa2l1	stefin A2 like 1	36.156811	5.14	19	3.70
Cstdc4	cystatin domain containing 4	36.184212	3.90	0	0.00
Csta3	cystatin A family member 3	36.210403	7.39	0	0.00
Csta2	cystatin A family member 2	36.221562	35.89	4	0.11
Stfa1	stefin A1	36.277148	8.22	10	1.22
Cstdc3	cystatin domain containing 3	36.305357	7.36	0	0.00
Cstdc6	cystatin domain containing 6	36.321665	12.67	9	0.71
Cstdc5	cystatin domain containing 5	36.359382	8.19	1	0.12
Stfa2	stefin A2	36.403946	4.42	3	0.68
Stfa3	stefin A3	36.450537	4.86	26	5.36
Casr	calcium-sensing receptor	36.490585	71.56	77	1.08
Cd86	**CD86 antigen**	36.568956	97.20	4	0.04
Ildr1	immunoglobulin-like domain containing receptor 1	36.693978	32.83	1	0.03
Slc15a2	**solute carrier family 15 (H+/peptide transporter), member 2**	36.750161	35.00	1	0.03
Eaf2	ELL associated factor 2	36.792884	82.18	7	0.09
Iqcb1	IQ calmodulin-binding motif containing 1	36.82836	44.36	5	0.11
Golgb1	golgi autoantigen, golgin subfamily b, macrogolgin 1	36.875093	57.99	9	0.16
