# Supplementary material for: Identification of susceptibility loci using a novel murine model for triple-negative breast cancer
Source: G3 (Bethesda). 2025 Oct 10;16(2):jkaf238. doi: 10.1093/g3journal/jkaf238 (PMC12869084; doi:10.1093/g3journal/jkaf238)
Supplement: jkaf238_Supplementary_Data [file jkaf238_supplementary_data.zip › Supplemental_Table_7_G3-2025-406194.pdf]

Supplemental Table 7. Chromosome 10 QTL Protein Coding Genes. An interval generated in GEMMA for chr10 (117.3-121.5 Mb) contains 121 annotated genes and open reading frames (ORFs), of which 27 were protein coding with gene symbol, description, start point, length, and variant or single nucleotide polymorphism (SNP) counts and density reported. Bolded genes are of particular interest.

Symbol	Gene Description	Mb Start	Length (Kb)	SNP Count	SNP Density
Cpsf6	cleavage and polyadenylation specific factor 6	117.344667	32.33	91	2.81
Cpm	carboxypeptidase M	117.6295	57.85	17	0.29
Mdm2	transformed mouse 3T3 cell double minute 2	117.688875	21.88	7	0.32
Slc35e3	solute carrier family 35, member E3	117.733678	12.68	1	0.08
Nup107	nucleoporin 107	117.750621	42.12	35	0.83
Rap1b	RAS related protein 1b	117.814597	31.38	9	0.29
Mdm1	transformed mouse 3T3 cell double minute 1	118.14154	27.46	68	2.48
Il22	interleukin 22	118.204584	5.46	0	0.00
Il22b	interleukin 22B	118.289629	5.41	1	0.18
Ifng	interferon gamma	118.441046	4.85	18	3.71
Ifngas1	Ifng antisense RNA 1	118.502035	54.49	59	1.08
Dyrk2	dual-specificity tyrosine-(Y)-phosphorylation regulated kinase 2	118.855602	33.64	29	0.86
Cand1	cullin associated and neddylation disassociated 1	119.198812	41.24	10	0.24
Grip1	glutamate receptor interacting protein 1	119.453238	634.03	1711	2.70
Grip1os2	glutamate receptor interacting protein 1, opposite strand 2	119.746062	15.64	130	8.31
Grip1os1	glutamate receptor interacting protein 1, opposite strand 1	119.825204	27.19	114	4.19
Helb	helicase (DNA) B	120.083608	29.40	29	0.99
Irak3	interleukin-1 receptor-associated kinase 3	120.141648	60.48	54	0.89
Tmbim4	transmembrane BAX inhibitor motif containing 4	120.208826	16.07	3	0.19
Llph	LLP homolog, long-term synaptic facilitation (Aplysia)	120.227048	5.02	0	0.00
Hmga2	high mobility group AT-hook 2	120.361268	115.20	56	0.49
Msrb3	methionine sulfoxide reductase B3	120.7811	117.91	95	0.81
Lemd3	LEM domain containing 3	120.923411	55.99	10	0.18
Wif1	Wnt inhibitory factor 1	121.034004	66.64	18	0.27
Tbc1d30	TBC1 domain family, member 30	121.263819	87.83	153	1.74
Gns	glucosamine (N-acetyl)-6-sulfatase	121.36509	32.16	111	3.45
Rassf3	Ras association (RalGDS/AF-6) domain family member 3	121.41035	65.90	207	3.14
