# Supplementary material for: Identification of susceptibility loci using a novel murine model for triple-negative breast cancer
Source: G3 (Bethesda). 2025 Oct 10;16(2):jkaf238. doi: 10.1093/g3journal/jkaf238 (PMC12869084; doi:10.1093/g3journal/jkaf238)
Supplement: jkaf238_Supplementary_Data [file jkaf238_supplementary_data.zip › Supplemental_Table_8_G3-2025-406194.pdf]

Supplemental Table 8. Tumor Histology Traits Display Insignificant Heritability in all BXD-BC Hybrids. Heritability (*h2*) and significance of strain effect (p) are shown tumor traits collected for N=26 BXD-BC crosses, with an average of 8 replicates per hybrid. Strain effect was tested by ANOVA. Hybrids that did not develop tumors were not included. Scored histology was averaged from over 3 randomly selected regions of interest in tumor H+E section.

Phenotype (Trait)	h2	p-value
Mitosis per high powered field (40X)	0.34	0.097
Vascularity and stroma	0.33	0.101
Necrosis	0.26	0.44
Epithelial Mesenchymal Transition (EMT)	0.24	0.557
