# Supplementary material for: Identification of susceptibility loci using a novel murine model for triple-negative breast cancer
Source: G3 (Bethesda). 2025 Oct 10;16(2):jkaf238. doi: 10.1093/g3journal/jkaf238 (PMC12869084; doi:10.1093/g3journal/jkaf238)
Supplement: jkaf238_Supplementary_Data [file jkaf238_supplementary_data.zip › Supplemental_Table_9_G3-2025-406194.pdf]

Supplemental Table 9. Chromosome 4 QTL Protein Coding Genes for Necrosis. An interval generated in GEMMA for chr4 (103.64-105.24 Mb) contains 23 annotated genes and open reading frames (ORFs), of which 6 were protein coding with gene symbol, description, start point, length, and variant or single nucleotide polymorphisms (SNP) counts and density reported.

Symbol	Gene Description	Mb Start	Length (Kb)	SNP Count	SNP Density
Dab1	disabled 1	103.619500	1125.34	1398	1.24
C8b	complement component 8, beta polypeptide	104.766317	38.23	0	0.00
C8a	complement component 8, alpha polypeptide	104.815679	60.81	2	0.03
Fyb2	FYN binding protein 2	104.835301	181.56	5	0.03
Prkaa2	protein kinase, AMP-activated, alpha 2 catalytic subunit	105.029649	80.25	141	1.76
Plpp3	phospholipid phosphatase 3	105.157347	75.42	49	0.65
